# Nanoscale Ferroelectric Characterization with Heterodyne Megasonic Piezoresponse Force Microscopy

**DOI:** 10.1002/advs.202003993

**Published:** 2021-02-15

**Authors:** Qibin Zeng, Hongli Wang, Zhuang Xiong, Qicheng Huang, Wanheng Lu, Kuan Sun, Zhen Fan, Kaiyang Zeng

**Affiliations:** ^1^ Department of Mechanical Engineering National University of Singapore Singapore 117576 Singapore; ^2^ The Key Lab of Guangdong for Modern Surface Engineering Technology National Engineering Laboratory for Modern Materials Surface Engineering Technology Institute of New Materials, Guangdong Academy of Science Guangzhou 510650 China; ^3^ MOE Key Laboratory of Low‐Grade Energy Utilization Technologies and Systems, School of Energy & Power Engineering Chongqing University Chongqing 400044 China; ^4^ Institute for Advanced Materials, South China Academy of Advanced Optoelectronics South China Normal University Guangzhou 510006 China; ^5^ Department of Electrical and Computer Engineering National University of Singapore Singapore 117583 Singapore; ^6^ NUS (Suzhou) Research Institute (NUSRI) Suzhou 215123 China

**Keywords:** CH_3_NH_3_PbI_3_ (MAPbI_3_) perovskite, electrostatic force, ferroelectric, heterodyne detection, high‐frequency excitation, piezoelectric, piezoresponse force microscopy

## Abstract

Piezoresponse force microscopy (PFM), as a powerful nanoscale characterization technique, has been extensively utilized to elucidate diverse underlying physics of ferroelectricity. However, intensive studies of conventional PFM have revealed a growing number of concerns and limitations which are largely challenging its validity and applications. In this study, an advanced PFM technique is reported, namely heterodyne megasonic piezoresponse force microscopy (HM‐PFM), which uses 10^6^ to 10^8^ Hz high‐frequency excitation and heterodyne method to measure the piezoelectric strain at nanoscale. It is found that HM‐PFM can unambiguously provide standard ferroelectric domain and hysteresis loop measurements, and an effective domain characterization with excitation frequency up to ≈110 MHz is demonstrated. Most importantly, owing to the high‐frequency and heterodyne scheme, the contributions from both electrostatic force and electrochemical strain can be significantly minimized in HM‐PFM. Furthermore, a special measurement of difference‐frequency piezoresponse frequency spectrum (DFPFS) is developed on HM‐PFM and a distinct DFPFS characteristic is observed on the materials with piezoelectricity. By performing DFPFS measurement, a truly existed but very weak electromechanical coupling in CH_3_NH_3_PbI_3_ perovskite is revealed. It is believed that HM‐PFM can be an excellent candidate for the ferroelectric or piezoelectric studies where conventional PFM results are highly controversial.

## Graphical abstract

A heterodyne megasonic piezoresponse force microscopy (HM‐PFM) is introduced for nanoscale ferro/piezoelectric characterization. HM‐PFM specially uses 10^6^ to 10^8^ Hz high‐frequency excitation and heterodyne method to measure piezoelectric strain, providing a substantial improvement for the signal source issue of conventional PFM. It is believed that HM‐PFM is an excellent candidate for ferro/piezoelectric studies where conventional PFM measurements are highly controversial.

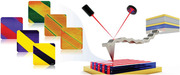

## 1 Introduction

With the growing demands of high density, miniaturization, and high integration for devices, micro‐ and nanoscale ferroelectric materials, phenomena, and devices have attracted extensive attentions from fundamental science to applications.^[^ ^1^, ^2^ ^]^ Although numerous theories have already been established for ferroelectrics at macroscale, many essential mysteries with respect to micro‐ or nanoscale ferroelectric behaviors, such as the polarization dynamics, domain growth kinetics, and the surface‐screening mechanisms, still remain ambiguous and pending to be elucidated.^[^ ^3^, ^4^ ^]^ Piezoresponse force microscopy (PFM), as an important branch of scanning probe microscopy (SPM), has evolved as the mainstream technique toward unveiling these underlying ferroelectric physics since its inception in 1992.^[^ ^2^, ^3^, ^4^, ^5^, ^6^ ^]^ In recent years, however, the extensive application of PFM has revealed a growing number of challenges and concerns about this technique and its interpretation, which is greatly challenging its validity in many ferroelectric studies recently.^[^ ^6^, ^7^, ^8^, ^9^, ^10^, ^11^, ^12^, ^13^ ^]^ Among these concerns, the signal source issue is pressingly pending to be addressed because the source of the signal is of fundamental importance for reaching correct interpretation of the PFM results.^[^ ^6^, ^7^, ^8^, ^13^ ^]^

Electrostatic force is one of the most intractable issues which continuously affect the PFM results since its invention. The existence of the electrostatic force can give rise to significant artifacts or misinterpretations in PFM‐based ferroelectric studies, resulting large numbers of ferroelectric‐like observations in many non‐ferroelectric materials.^[^ ^6^, ^7^, ^8^, ^14^, ^15^, ^16^, ^17^, ^18^, ^19^ ^]^ Meanwhile, due to the pronounced contribution of the electrostatic force, researchers usually measure the ferroelectric hysteresis loop by using the pulsed DC method in which the electrostatic force effect can be minimized but the polarization back‐switching effect is involved inevitably.^[^ ^3^, ^15^, ^20^, ^21^ ^]^ Therefore, a large amount of research work has been implemented to eliminate or quantify the electrostatic force contribution in PFM measurements.^[^ ^2^, ^8^, ^14^, ^15^, ^16^, ^17^, ^22^, ^23^, ^24^, ^25^, ^26^, ^27^, ^28^, ^29^, ^30^, ^31^, ^32^ ^]^ It has been proved that the electrostatic contribution can be relatively minimized by various methods, such as imaging the materials with strong piezoelectricity, using stiff or shielded probes and applying DC compensation voltage.^[^ ^8^, ^15^, ^21^, ^23^, ^25^, ^26^, ^27^, ^32^ ^]^ Furthermore, considering the difficulties in technically eliminating the electrostatic force, off‐line analysis strategies or complementary experiments (such as the contact Kelvin probe force microscopy) are proposed to quantify the electrostatic force contribution thus achieving a correct interpretation of the PFM results.^[^ ^14^, ^29^, ^33^ ^]^ However, each of these methods is subjected to specific limitations,^[^ ^16^, ^27^, ^28^, ^32^, ^34^, ^35^ ^]^ and so far researcher are still paying large effort to raise an ideal approach which can eliminate the electrostatic force contribution while causing negligible influence to detect the real piezoelectric responses. In addition, in some materials, the electrochemical Vegard strain caused by the diffusion and electromigration of the mobile ions can also affect the PFM measurements significantly.^[^ ^7^, ^8^, ^16^ ^]^ Due to the similarity of the electrochemical and piezoelectric strains, it is difficult to differentiate the electrochemical and piezoelectric signals especially when the property of the sample is unknown beforehand.^[^ ^6^, ^17^, ^36^, ^37^ ^]^ Although attentions have been paid by researchers to identify the true PFM signal,^[^ ^7^, ^37^, ^38^ ^]^ these approaches are restricted by the materials’ property and the co‐existence of electrostatic force during the measurements.^[^ ^14^, ^15^, ^16^ ^]^ By far, attributing the signal source of the PFM to piezoelectric or electrochemical strains mainly relies on the material's property, and the effective ways to reduce the influence of electrochemical strain are still pending to be explored.^[^ ^17^ ^]^ Except for the electrostatic force and electrochemical strain, the electrostrictive coupling also contributes to the PFM signal.^[^ ^37^, ^39^, ^40^ ^]^ Since the electrostriction shows a quadratic relationship with respect to the applied electric field,^[^ ^41^, ^42^ ^]^ its influence on PFM is similar to that of the electrostatic force. Moreover, there may be multiple other electric field‐induced effects which can directly or indirectly induce responses in PFM measurements, including electrochemical dipoles, charge injection, field effect, electrochemical reactions, flexoelectricity and Joule heating and so on.^[^ ^6^, ^7^, ^8^, ^19^, ^22^, ^37^, ^40^, ^42^, ^43^, ^44^, ^45^, ^46^ ^]^ Obviously, the issue on signal sources causes large complexity and uncertainty in current PFM measurements, which greatly challenges its application especially for the materials with unknown properties. For example, the ongoing hot debate about the ferroic nature of methylammonium lead triiodide (CH_3_NH_3_PbI_3_ or MAPbI_3_) perovskite is such a striking illustration of the challenges faced by conventional PFM, since the true origin of the PFM signal observed on MAPbI_3_ is remaining unclear.^[^ ^9^, ^10^, ^11^, ^12^, ^47^, ^48^, ^49^, ^50^, ^51^, ^52^, ^53^ ^]^ In brief, even though great effort has been made to purify the PFM signal, the inherent complexity of electrochemical coupling phenomena still pressingly requires further substantial progresses to achieve an ideal PFM measurement.

Recently, high‐frequency PFM working at high eigenmodes of the cantilever has been put forward to effectively minimize the electrostatic force contribution.^[^ ^24^, ^26^, ^30^ ^]^ However, the associated decrease of detection sensitivity, laser spot size effect, large bandwidth requirement for photodetector and lock‐in amplifier make the application of high‐frequency PFM very limited.^[^ ^24^, ^30^ ^]^ Despite high‐frequency PFM has seldom received attentions due to the current technical restrictions, using high‐frequency excitation to detect piezoelectric strain does provide a meaningful instruction for the development of advanced PFM. In this study, by performing an overall assessment of using high‐frequency excitation in PFM, it is found that several substantial improvements can be achieved simultaneously, including minimizing the electrostatic force‐induced cantilever vibration, attenuating electrochemical Vegard strain and electrostriction effects, as well as reducing the influence of dynamic electrochemical processes. Most importantly, to effectively break the technical limitations and achieving PFM measurements at much higher frequency, heterodyne detection scheme has been introduced to PFM. Based on using high‐frequency excitation and heterodyne detection, an advanced PFM technique which focuses on detecting high‐frequency piezoelectric strain is developed here. This technique, that we named as heterodyne megasonic piezoresponse force microscopy (HM‐PFM),^[^ ^54^ ^]^ utilizes both electrical and mechanical drives with MHz frequency to probe the surface piezoelectric vibration at nanoscale via heterodyne detection method. Conventional ferroelectric materials are first used to test the basic ferroelectric domain characterization, switching spectroscopy, and high‐frequency operation capabilities of the HM‐PFM. The influence of electrostatic force in the HM‐PFM measurement is systematically studied and compared with the results from conventional PFM. The results unambiguously reveal that HM‐PFM can obtain ideal ferroelectric domain image, standard ferroelectric hysteresis loop and operate up to as high as ≈110 MHz, at the same time, the electrostatic force contribution has been significantly minimized. Finally, the special measurement offered by HM‐PFM, the difference‐frequency piezoresponse frequency spectrum (DFPFS),^[^ ^54^ ^]^ is demonstrated on three types of functional materials including dielectric, lithium‐ion battery and ferroelectric materials. The DFPFS results do show a distinct difference between the piezoelectric and non‐piezoelectric materials tested here, and at the same time, indicate that the electrochemical strain has been considerably attenuated in HM‐PFM. Furthermore, with the DFPFS measurement, a truly existed but very weak electromechanical coupling is observed in MAPbI_3_ perovskite.

## 2 Results and Discussion

### 2.1 High‐Frequency Excitation

In this new designed HM‐PFM, the excitation frequency for tip and sample are both located within high‐frequency region, typically 10^6^ to 10^8^ Hz. As mentioned earlier, operating PFM at high frequency does provide substantial improvement for numbers of signal source issues, which include minimizing the electrostatic force‐induced cantilever vibration, attenuating electrochemical Vegard strain and electrostriction effects, as well as reducing the influence of dynamic electrochemical processes, and below are the detailed analyses.

The electrostatic force‐induced cantilever vibration can be significantly minimized when sample is excited by high‐frequency electric filed. For the commonly used free rectangular AFM cantilever, the flexural vibration in air can be described by the classical Euler–Bernoulli beam theory, the effective force constant *k_n_* and quality factor *Q_n_* of *n*th eigenmode are respectively given by (ignoring the internal damping)^[^ ^55^ ^]^

(1) $$k_{n}=\frac{\xi_{n}^{4}}{4}\frac{EI}{L^{3}}$$

(2) $$Q_{n}=\frac{\sqrt{mEI}}{c}{\left(\frac{\xi_{n}}{L}\right)}^{2}$$

where *E*, *I*, *L* and *m* are Young's modulus, moment of inertia, length, and mass per unit length of the cantilever, respectively; *c* is the hydrodynamic damping coefficient and *ξ*_*n*_ is the wave numbers (*ξ*_1_ = 1.875, *ξ*_2_ = 4.694…) of the infinite flexural vibration modes which increases linearly with the mode number.^[^ ^56^ ^]^ Equations (1) and (2) indicate that the effective force constant *k* will increase with the fourth power of the mode number, while quality factor *Q* only increase quadratically with the mode number (Figure S1, Supporting Information). Therefore, even ignoring the internal damping, the excitation of high eigenmodes will become more and more difficult. A detailed calculation for the amplitude response of AFM cantilever as a function of excitation frequency has been performed and presented in Section S1 in the Supporting Information. The results clearly show that the oscillation amplitude decays dramatically with increasing frequency, implying that the electrostatic force induced cantilever vibration will be significantly minimized if PFM is operated at high frequency.

With high‐frequency excitation, the contribution from the electrochemical Vegard strain to PFM results can also be largely reduced. The Vegard strain is resulted from the tip voltage‐induced diffusion and electromigration of mobile ions. Since the motion of ion is highly frequency dependent, it is possible to change the magnitude of Vegard strain by changing the excitation frequency. In a typical PFM operation frequency range (≈300 kHz), the relationship between Vegard strain caused surface displacement and tip voltage frequency is employed as follows^[^ ^38^, ^57^ ^]^

(3) $$u_{3}=\frac{2\left(1+v\right)\beta \sqrt{D}}{\eta }\frac{V_{\mathrm{ac}}}{\sqrt{\omega }}$$

where *u*_3_, *ν*, *β*, *D*, *V*_ac,_ and *ω* are the amplitude of the surface displacement, Poisson's ratio, effective Vegard coefficient, the diffusion coefficient of the ion, amplitude, and frequency of AC voltage, respectively, and *η* represents the linear relation between chemical potential and applied electric field. According to Equation (3), the surface displacement is proportional to the reciprocal of the square root of the frequency, implying that the Vegard strain effect can be effectively suppressed at high frequency.^[^ ^17^, ^58^ ^]^ Furthermore, if the modulation frequency is very high, i.e., far higher than the cut‐off frequency of the ionic diffusion, the electrochemical Vegard strain is expected to become negligible, as the ions cannot diffuse as fast as the applied AC voltage (i.e., the ions are in a quasi‐static state).^[^ ^17^, ^58^, ^59^ ^]^ Both theoretical and experimental results have already demonstrated the attenuation of Vegard strain with increasing frequency for several lithium‐containing materials.^[^ ^60^, ^61^ ^]^

In addition, increasing the frequency of probing wave can decrease the influences from electrostriction and dynamic electrochemical processes in the PFM measurement as well. Considering the deformation along the direction of applied electric field *E*_elec_ = *E*_0_ + *E*_ac_sin(*ωt*), the electrostriction strain is *S* = *Q*_es_(*P*_0_ + *εE*_elec_)^2^ and the first‐harmonic strain which contributes to the piezoresponse signal is *S*_*ω*_ = 2*Q*_es_(*εP*_0_ *E*_ac_ + *ε^2^E*_0_ *E*_ac_)sin(*ωt*), where *Q*_es_ is the polarization related electrostrictive coefficient, *P*_0_ is an static net polarization (e.g., spontaneous polarization) and *ε* is the dielectric constant.^[^ ^40^, ^41^ ^]^ It has been found that the electrostrictive effect is usually governed by the dielectric constant instead of electrostrictive coefficient, and large electrostriction mainly exists in the materials with pretty large dielectric constant.^[^ ^62^ ^]^ According to the Debye relaxation‐based dielectric dispersion relationship, the dielectric constant (real part) will decrease with increasing frequency.^[^ ^63^ ^]^ Therefore, for those materials with an obvious dielectric dispersion in the frequency range of less than 10^6^ Hz,^[^ ^63^, ^64^ ^]^ increasing the frequency of the electric field to 10^6^–10^8^ Hz, the electrostrictive strain is expected to attenuate due to the decrease of dielectric constant. Furthermore, some other dynamic electrochemical processes, such as the formation of defects under the sample surface, water‐splitting reaction, nano‐oxidation, surface redox reaction and certain unusual electrochemical phenomena may also affect the PFM measurement especially in the ambient environment.^[^ ^7^, ^44^, ^45^, ^65^, ^66^ ^]^ Typically, the majority of these electrochemical processes are dominated by the motion of the ions, defects, vacancies, etc. Since the motion of these ions and defects has a specific temporal scale, it is safe to predict that once the motion of the ions and defects cannot follow the variation of the drive voltage, the associated dynamic electrochemical processes will be largely suppressed.^[^ ^44^, ^60^ ^]^

### 2.2 Heterodyne Detection

As the direct high‐frequency detection is subjected by multiple limitations as discussed previously, we therefore introduce an indirect scheme, the heterodyne detection, to break those limitations. Heterodyne detection is an extensively used method in the measurement and analysis of high‐frequency signals. The heterodyne process is to down‐convert the high‐frequency signal to a lower, easily measurable frequency by mixing it with a known reference signal.^[^ ^67^ ^]^ Due to the nonlinear nature of tip‐sample interaction, the heterodyne detection scheme has been successfully used in multi‐frequency SPM, especially the ultrasound‐based AFM where direct high‐frequency detection is usually difficult or impractical.^[^ ^67^, ^68^, ^69^ ^]^ To measure the high‐frequency ultrasonic vibration of sample by heterodyne method, the tip is usually excited to vibrate at a slightly different frequency (i.e., serve as the reference vibration), when tip‐sample interaction force is highly nonlinear (such as in contact mode), there will be a mechanical frequency mixing process thus a difference‐frequency force component is generated and the sample's high‐frequency vibration can be detected from this difference‐frequency component.^[^ ^67^, ^68^, ^69^ ^]^ Similarly, if a high‐frequency reference vibration is introduced in PFM, the sample's high‐frequency piezoelectric vibration can thus be detected via the mechanical frequency mixing process. In the designed HM‐PFM here, the reference vibration is provided by mechanically driving the cantilever via the holder transducer, i.e., the tip has a mechanical vibration in addition to the sample's piezoelectric vibration. Considering the tip and sample's vibration by *A*_t_sin(2*πf*_t_ *t* + *ϕ*_t_) and *A*_s_in(2*πf*_s_ *t* + *ϕ*_s_) respectively (Figure S3, Supporting Information), the time‐dependent tip‐sample interaction force now is given by

(4) $$F_{\mathrm{ts}}\left(z,t\right)=F_{\mathrm{ts}}\left[z_{0}+A_{t}\sin\left(2\pi f_{t}t+\phi_{t}\right)-A_{s}\sin\left(2\pi f_{s}t+\phi_{s}\right)\right]$$

where *z* and *z*_0_ are the instantaneous and equilibrium tip‐sample separations, respectively. Since the tip‐sample interaction force *F*_ts_ depends nonlinearly with *z*, under the condition of small vibration amplitudes, *F*_ts_ can be approximately expressed by a Taylor series at *z* = *z*_0_ up to second order

(5) $$F_{\mathrm{ts}}\left(z,t\right)\approx F_{\mathrm{ts}}\left(z_{0}\right)+F_{\mathrm{ts}}^{\prime }\left(z_{0}\right)\left(z-z_{0}\right)+\frac{1}{2}F_{\mathrm{ts}}^{\prime \prime }\left(z_{0}\right){\left(z-z_{0}\right)}^{2}$$

Combining Equations (4) and (5) and ignoring all the static and high‐frequency items, the difference‐frequency force is given by (see Section S2 in the Supporting Information)

(6) $$F_{\mathrm{ts}}{\left(z,t\right)}_{\mathrm{diff}}=-\frac{1}{2}F_{\mathrm{ts}}^{\prime \prime }\left(z_{0}\right)A_{t}A_{s}\cos\left(2\pi f_{\mathrm{diff}}t+\phi_{s}-\phi_{t}\right)$$

in which *f*_diff_ = *f*_s_ − *f*_t_. If the drive frequencies of tip and sample are set to be close, the difference‐frequency force *F*_ts_(*z*)_diff_ will be located at low‐frequency range and can normally drive the cantilever to oscillate and then detected by conventional SPM setup, even though very high frequencies are used. For the purpose of ferroelectric characterization, revealing the contrasts of sample vibration amplitude *A*_s_ and phase *ϕ*_s_ are necessary because the information of interest (such as domain wall and polarization direction) is included in *A*_s_ and *ϕ*_s_. From Equation (6), it is clear to see if the second‐order force gradient, tip vibration amplitude and phase are constant, the sample vibration amplitude and phase can be extracted via detecting the difference‐frequency cantilever vibration (see more details in Section S2 in the Supporting Information). As the tip always keeps a constant force on sample surface during contact scanning, the above‐mentioned requirements can be achieved especially when scanning area is relatively flat and uniform. Therefore, by using heterodyne detection, the limitations of the previously mentioned high‐frequency excitation have been removed, and the characterization of ferroelectric domain with very high excitation frequency now becomes possible (as the ≈110 MHz achieved in this study). However, HM‐PFM is based on contact AFM mode, just as the conventional PFM, the tip‐sample contact properties will also make significant influences to the final piezoresponse signal.^[^ ^7^, ^32^, ^70^ ^]^ Therefore, like other contact mode‐based SPM techniques, to achieve a more unambiguous mapping result, it is always suggested to operate the HM‐PFM at a tip‐sample contact status as constant as possible, such as selecting a flat area by using tapping AFM mode before conducting HM‐PFM measurement (the configuration of HM‐PFM does well support the seamless switching between the tapping mode AFM and HM‐PFM), decreasing the scanning size if possible and avoiding directly scanning a quite rough surface without any roughness optimizing processes.

### 2.3 Experimental Setup

**Figure 1:**
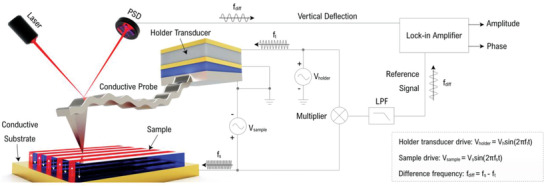
Schematic diagram of HM‐PFM. The diagram schematically shows the setup of the model HM‐PFM system (not on scale). Tip is mechanically driven by the holder transducer with a frequency *f*_t_, while the sample is electrically driven by the electric field between grounded tip and conductive substrate with a frequency *f*_s_. The DFP signal generated from the nonlinear tip‐sample interaction has a frequency of *f*_diff_ = *f*_s_ − *f*_t_.

In this study, the model HM‐PFM system is constructed on a commercial SPM system (SPA400, Seiko Instruments Inc., Japan). The complete setup of this model system is depicted in **Figure** **1**. In the HM‐PFM designed here, tip is set to be grounded while the drive signal for stimulating electromechanical strain is sent to the conductive substrate of the sample. In order to mechanically drive the cantilever at high frequency, the original probe holder is specially modified to enable an effective external high‐frequency excitation. During the measurement, the tip vibration is stimulated by the holder transducer via holder drive *V*_holder_ at frequency *f*_t_, while and the sample vibration is excited by the sample drive *V*_s_ at frequency *f*_s_. The difference‐frequency oscillation (here we name it as difference‐frequency piezoresponse, DFP) generated from the heterodyne process is detected by the position sensitive detector (PSD) and then demodulated by the lock‐in amplifier. A home‐made analog multiplier and a low‐pass filter (LPF) are used to produce difference‐frequency reference signal for coherent demodulation. Alternatively, the reference signal can be provided internally by synchronizing the clocks of signal source and lock‐in amplifier.^[^ ^54^ ^]^ The demodulation results, i.e., amplitude and phase, of the DFP signal are sent to the controller of AFM and then synchronously imaged with the topography (all the amplitude images here are shown in dimension of a.u.). In order to enhance the signal‐to‐noise ratio (SNR) of the DFP signal, the tip drive frequency *f*_t_ is typically set to close the eigenfrequency of a high eigenmode (typically in MHz range) to generate a stronger vibration; and the difference frequency *f*_diff_ is usually set to a value near the first or second‐order contact resonance frequency of the cantilever, where the resonance amplification can be utilized;^[^ ^68^ ^]^ then the sample drive frequency *f*_s_ is set to be *f*_t_ + *f*_diff_. The determination of *f*_t_ and *f*_s_ are completed via a self‐developed frequency‐selecting program which typically works within 1 to 200 MHz. To obtain a better detection sensitivity and decrease the tip‐sample interaction force thus reducing the tip and surface damages, it is recommended to use cantilevers with relatively small stiffness (such as 0.2–2 N m^−1^ in this study) in HM‐PFM. As contact resonance amplification is used in HM‐PFM, which is similar to the conventional PFM operated in contact resonance,^[^ ^70^ ^]^ the tip‐sample contact property becomes important during the measurement and attentions should be paid to keep a constant tip‐sample contact status during scanning via the above‐mentioned methods.

### 2.4 Ferroelectric Domain Characterization and Switching Spectroscopy

**Figure 2:**
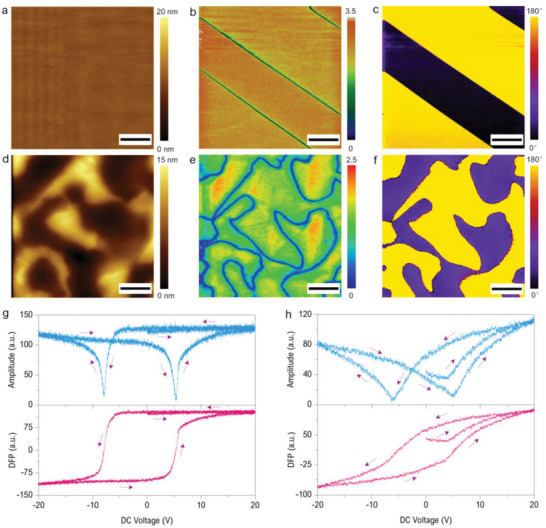
Ferroelectric domain characterization and hysteresis loop measurement. a) Topography, b) HM‐PFM amplitude image, and c) HM‐PFM phase image of the PPLN sample. d) Topography, e) HM‐PFM amplitude image, and f) HM‐PFM phase image of the PZN‐9%PT sample. g) Hysteresis loop of PZT film and h) PZN‐9%PT measured by HM‐PFM switching spectroscopy using the continuous DC method. Blue and red curves are amplitude and DFP [= amplitude × cos(phase)] as a function of applied DC voltage, respectively, and the arrows denote the direction of the loops. Measurement conditions: b,c) *f*_t_ = 8.356 MHz and *f*_s_ = 8.63959 MHz; e,f) *f*_t_ = 8.395 MHz and *f*_s_ = 8.6813 MHz; g) *f*_t_ = 13.516 MHz and *f*_s_ = 13.82158 MHz; h) *f*_t_ = 15.451 MHz and *f*_s_ = 15.75504 MHz. Scale bar in (a–f), 2 µm, image size = 10 × 10 µm^2^.

In this study, the periodically poled lithium niobate (PPLN) single crystal is used as the standard test sample, similar to other PFM characterization.^[^ ^34^, ^71^ ^]^ **Figure** **2**a–c shows the typical scanning results of the PPLN by HM‐PFM with the operating frequencies *f*_t_ = 8.356 MHz and *f*_s_ = 8.63 959 MHz (*f*_diff_ = 283.59 kHz) whereas the other scanning parameters are nearly the same with that used in conventional PFM.^[^ ^70^ ^]^ From the amplitude and phase images of the DFP signal, the domain walls between the two adjacent domains can be clearly observed in the amplitude image (Figure 2b), and the periodical domains with alternative upward and downward polarization are distinctly revealed in the phase image (Figure 2c). Meanwhile, Figure 2b,c indicates a uniform amplitude distribution and a nearly 180° phase difference between the domains with opposite polarization, which agrees well with the characteristics of the proposed “ideal” PFM measurements of ferroelectric materials.^[^ ^34^ ^]^ Followed by this, another ferroelectric material, Pb(Zn_1/3_Nb_2/3_)O_3_‐9%PbTiO_3_ (PZN‐9%PT) single crystal, is studied here to further test the HM‐PFM. The PZN‐9%PT sample tested here has spontaneously polarized domains in which the polarization is along thickness direction, and the upward and downward domains are randomly distributed.^[^ ^72^ ^]^ Typical topography and simultaneously obtained HM‐PFM results of the PZN‐9%PT sample are shown in Figure 2d–f, where the operation frequencies are: *f*_t_ = 8.395 MHz and *f*_s_ = 8.6813 MHz (*f*_diff_ = 286.3 kHz). Clear labyrinthine domain pattern and ≈180° phase difference between the upward and downward domains can be observed, which are highly consistent with the reported results,^[^ ^72^, ^73^ ^]^ thereby once again confirming the validity of HM‐PFM in the ferroelectric domain characterization. As mentioned above, HM‐PFM results may be affected by the tip‐sample contact status, here a coarsely polished PZN‐9%PT sample has been characterized to show the situation, and the results are displayed in Figure S10 in the Supporting Information. It can be seen that with a surface roughness of ≈10 nm here, the HM‐PFM can still define the domain structure clearly though topography cross‐talk is pronounced. However, when scanning those very rough surfaces, due to the dramatic variation of tip‐sample contact status, there will exist a significant topography cross‐talk in the amplitude and phase images which may affect the final conclusion. Therefore, similar with the conventional resonance enhanced PFM,^[^ ^70^ ^]^ much more careful analysis should be performed when using HM‐PFM to map the rough samples. In addition, a continuously repeated scanning experiment has been implemented to demonstrate that the HM‐PFM amplitude and phase result is negligibly affected by the change of tip‐sample contact radius (Section S8, Supporting Information).

The measurement of hysteresis loop by using HM‐PFM switching spectroscopy is further performed here. The switching spectroscopy in HM‐PFM is similar with that from conventional PFM.^[^ ^15^, ^20^, ^54^ ^]^ A continuous or pulse triangular wave‐like DC bias sequence is superimposed on the AC drive and then applied to the sample to induce the polarization switching, while at the same time, measuring the DFP signal as a function of DC bias. Two ferroelectric materials are measured here, the first one is 300 nm Pb(Zr,Ti)O_3_ (PZT) film, and the other is PZN‐9%PT. A continuous triangular DC probing wave with a step duration of 1 ms is employed to measure the hysteresis loops, which is similar with the macroscale polarization‐electric field hysteresis loop measurement.^[^ ^74^ ^]^ Figure 2g,h displays the attained hysteresis loops of PZT and PZN‐9%PT, respectively. Both the HM‐PFM amplitude loops (blue curves) of the PZT (Figure 2g) and PZN‐9%PT (Figure 2h) samples show the expected butterfly shape which is the common characteristic of the ferroelectricity.^[^ ^7^, ^14^, ^74^ ^]^ Meanwhile, the DFP (red curves), calculated by amplitude × cos(phase), on both PZT (Figure 2g) and PZN‐9%PT (Figure 2h) samples manifest the typical ferroelectric hysteresis with applied DC bias.^[^ ^7^, ^14^, ^74^ ^]^ Note that, instead of using the conventional pulsed DC method to minimize the electrostatic force effect in the conventional PFM,^[^ ^3^, ^15^, ^20^, ^21^ ^]^ the continuous DC method is employed in HM‐PFM to acquire hysteresis loops. It is obvious that the obtained results (Figure 2g,h) have no observable features of the effects of electrostatic force as those in the conventional PFM^[^ ^14^, ^15^, ^75^ ^]^ though a soft cantilever (0.2–2.0 N m^−1^) is used, this improvement in fact benefits from the HM‐PFM's powerful electrostatic force suppression capability which will be discussed later. In brief, the results shown in Figure 2g,h well demonstrates that HM‐PFM switching spectroscopy can provide standard hysteresis loop measurement for the study of local polarization dynamics.

### 2.5 High‐Frequency Operation

**Figure 3:**
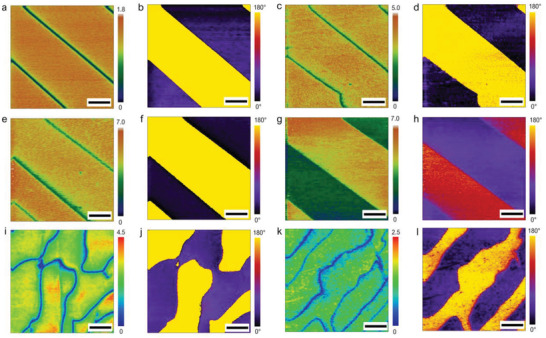
Ferroelectric domain characterization with high‐frequency excitation. a,c,e,g) HM‐PFM amplitude image and b, d, f, h) the respective HM‐PFM phase image of PPLN sample. i,k) HM‐PFM amplitude image and j,l) the respective HM‐PFM phase image of PZN‐9%PT sample. Measurement conditions: a,b) *f*_t_ = 27.9 MHz and *f*_s_ = 28.1882 MHz; c,d) *f*_t_ = 40.195 MHz and *f*_s_ = 40.4823 MHz; e,f) *f*_t_ = 62.255 MHz and *f*_s_ = 62.61 MHz; g,h) *f*_t_ = 108.95 MHz and *f*_s_ = 109.137 MHz; i,j) *f*_t_ = 27.871 MHz and *f*_s_ = 28.13966 MHz; k,l) *f*_t_ = 42.9 MHz and *f*_s_ = 42.975 MHz. Scale bar in (a–g), 2 µm and image size = 10 × 10 µm^2^; scale bar in (i–l), 1 µm and image size = 5 × 5 µm^2^.

As the HM‐PFM uses the heterodyne method to detect the piezoelectric strain, the excitation frequency for sample now is no longer limited by the bandwidth of conventional optical lever system. According to the advantages provided by high‐frequency excitation, one can expect a more unambiguous measurement for piezoelectric or ferroelectric information if higher frequencies are used. Herein, PPLN and PZN‐9%PT are still used to explore the high‐frequency detection capability of the HM‐PFM. **Figure** **3** displays the amplitude and phase images of the DFP signal with drive frequencies ranging from ≈30 to ≈110 MHz. Without loss of generality, each pair of amplitude and phase images are obtained by using different tips and scanning on different areas (including both clean and contaminated areas). From the results of PPLN shown in Figure 3a–f, clear domain wall, uniform amplitude distribution and a near 180° phase difference between the domains with opposite polarization can be observed under the excitation frequencies of *f*_s_ = 28.1882 MHz (Figure 3a,b), 40.4823 MHz (Figure 3c,d), and 62.61 MHz (Figure 3e,f). Similar results can also be observed on PZN‐9%PT sample, which are shown in Figure 3i,j (*f*_s_ = 28.13966 MHz) and Figure 3k,l (*f*_s_ = 42.975 MHz). Surprisingly, it is found that an effective ferroelectric domain characterization for PPLN can still be achieved even when the drive frequency is increased to *f*_s_ = 109.137 MHz (Figure 3g,h). Although the amplitude and phase images shown in Figure 3g,h display a deviation from the “ideal” PFM measurements,^[^ ^71^ ^]^ the periodical domain structure are still presented unambiguously. The deviation at near 110 MHz indicates that a background signal with the same frequency of *f*_diff_ gets involved into the DFP signal, thus causing an obvious amplitude contrast and a non‐180° phase difference between the upward and downward domains.^[^ ^76^ ^]^ This background signal actually comes from the radio‐frequency radiation effect due to the ≈110 MHz high frequency and imperfect electromagnetic shielding of the holder transducer. If the holder transducer can be well electromagnetically shielded, this background signal will be minimized (see section S6 in the Supporting Information). However, even under the influence of radio‐frequency radiation, the domain structure of PPLN can still be clearly revealed by our model HM‐PFM system with ordinary AFM probes at the excitation frequency up to ≈110 MHz. It is still possible to further improve the operation frequency by optimizing the model HM‐PFM system, such as enhancing electromagnetic shielding, using special AFM probes^[^ ^77^ ^]^ and increasing the center frequency of the holder transducer, etc. To our best knowledge, this ≈110 MHz is about 100 to 1000 times higher than the frequency used in the conventional PFM and more than ten times higher than the highest excitation frequency (8.4 MHz^[^ ^30^ ^]^) achieved in other reported high‐frequency PFM previously.

### 2.6 Electrostatic Force Contribution

**Figure 4:**
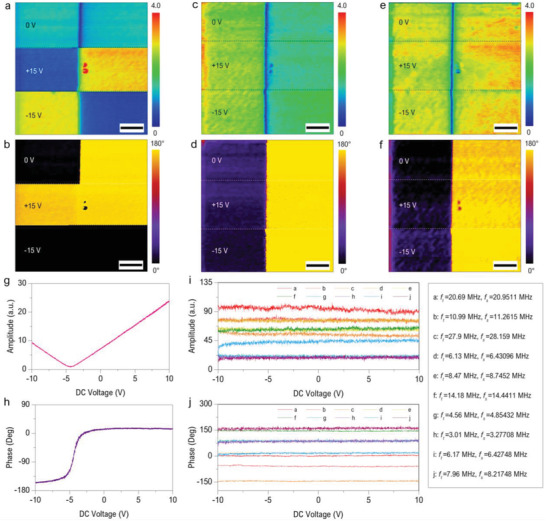
Electrostatic force contribution in the measurement of PFM and HM‐PFM on PPLN. a) Single‐frequency PFM amplitude image and b) phase image with 0, +15, and −15 V DC bias applied. c,e) HM‐PFM amplitude image and d,f) the respective HM‐PFM phase image with 0, +15, and −15 V DC bias applied. g) Typical single‐frequency PFM amplitude and h) phase as a function of DC voltage. i) HM‐PFM amplitude and j) phase as a function of DC voltage under various excitation frequencies (measured at the same position with (g,h)). Measurement conditions: c,d) *f*_t_ = 8.14 MHz and *f*_s_ = 8.422 MHz; e,f) *f*_t_ = 7.176 MHz and *f*_s_ = 7.45915 MHz. Scale bar in (a–f), 500 nm and image size = 3 × 3 µm^2^.

One of the most important considerations concerning using high frequency in PFM is to minimize the electrostatic force contribution. Therefore, the contribution of the electrostatic force in the piezoresponse signal of HM‐PFM is carefully investigated and compared with that from the conventional PFM. PPLN is still selected as the test sample, as it is reported that its PFM measurements can be affected by electrostatic force significantly.^[^ ^23^, ^24^, ^25^ ^]^ When the sample drive voltage *V*_s_sin(2*πf*_s_ *t*) with a DC bias *V*_dc_ is applied between the tip and the sample, the resultant electrostatic force *F*_EF_ is *C*′[*V*_dc_ − *V*_cpd_ + *V*_s_sin(2*πf*_s_ *t*)]^2^/2, where *C*′ is the capacitance gradient of tip‐sample capacitor and *V*_cpd_ is the contact potential difference between tip and sample surface. In the conventional PFM measurements, the piezoelectric strain signal is demodulated from the first‐harmonic response, thus the first‐harmonic component of the electrostatic force, *F*_EF‐*ω*_ = *C*′(*V*_dc_ − *V*_cpd_)*V*_s_sin(2*πf*_s_ *t*), will naturally get involved into the demodulation result. Obviously, *F*_EF‐*ω*_ varies with the applied DC bias, so that the contribution of electrostatic force in the conventional PFM can be revealed by changing the DC bias. **Figure** **4**a,b shows the single‐frequency PFM amplitude and phase images of the PPLN under DC biases of 0 and ±15 V. It is evident that when DC bias is 0 V, the expected standard amplitude and phase contrasts can be obtained. However, when DC bias is changed to ±15 V, striking amplitude contrast appeared and only slight phase difference can be observed between the domains with opposite polarization. Since ±15 V bias will not cause polarization switching due to the high coercive field of the PPLN,^[^ ^78^ ^]^ this phenomenon is ascribed to the significant contribution from the electrostatic force.^[^ ^23^, ^24^, ^25^ ^]^ To make the comparison, exactly the same experiment is performed in situ by using HM‐PFM and the results are displayed in Figure 4c–f. In comparison to the significant change of amplitude and phase contrasts in the single‐frequency PFM measurement, the results obtained by HM‐PFM under two different operating frequencies (*f*_s_ = 8.422 MHz in Figure 4c,d and *f*_s_ = 7.45 915 MHz in Figure 4e,f) show almost no change when ±15 V DC biases are applied. To further examine the dependence of amplitude and phase with varying DC bias, the same DC spectroscopy experiments are performed at the same position by single‐frequency PFM and HM‐PFM. The amplitude and phase as a function of DC voltage measured by scanning the DC bias within ±10 V in single‐frequency PFM are shown in Figure 4g,h, respectively. Obviously, the V‐shaped amplitude curve and near 180° phase change indicate that the first‐harmonic electrostatic force is affecting the piezoresponse signal of the PPLN sample significantly. By contrast, completely different variation trends of the amplitude and phase can be observed from the measurement using the HM‐PFM (Figure 4i,j). It is evident that, even using various excitation frequencies, almost all of the amplitude and phase signals keeps a constant magnitude with the changing DC bias. The same experiments have also been conducted on X‐cut quartz single crystal (0.5 mm thick) where the piezoelectricity is weak and the electrostatic force contribution can be prominent.^[^ ^18^ ^]^ Despite the weak piezoelectricity, the results still show a similar constant trend (Figure S6, Supporting Information), which strongly indicates that the contribution from the electrostatic force in HM‐PFM measurement has been significantly minimized. Therefore, the hysteresis loops (Figure 2g,h) measured by using continuous DC method with a soft cantilever can manifest neglectable electrostatic force effects.

**Figure 5:**
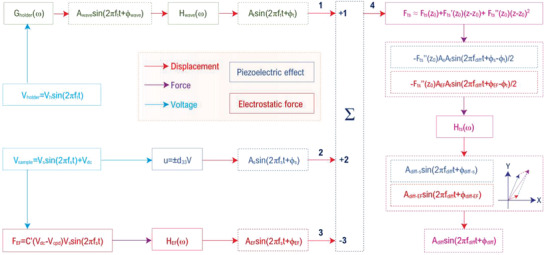
Schematic of DFP signal generation mechanism in HM‐PFM with electrostatic force. **1** Mechanically excited tip vibration *A*_t_sin(2*πf*_t_ *t* + *ϕ*_t_), **2** sample piezoelectric vibration *A*_s_sin(2*πf*_s_ *t* + *ϕ*_s_), **3** electrostatic force excited tip vibration *A*_EF_sin(2*πf*_s_ *t* + *ϕ*_EF_), and **4** instantaneous tip‐sample separation (**4** = **1** + **2** − **3**). *G*(*ω*) is the transfer function of holder transducer and *u* = ±*d*_33_ *V* represents the linear piezoelectric effect. *H*_wave_(*ω*), *H*_EF_(*ω*) and *H*_ts_(*ω*) are cantilever transfer functions under the excitations of mechanical wave, electrostatic force, and local tip‐sample interaction, respectively. The *X*–*Y* plane schematically depicts the vectorial synthesis of *A*_diff‐s_sin(2*πf*_diff_ *t* + *ϕ*_diff−s_) and *A*_diff‐EF_sin(2*πf*_diff_ *t* + *ϕ*_diff‐EF_).

The mechanism of minimizing electrostatic force contribution in HM‐PFM can be understood from the piezoresponse signal generation process shown in **Figure** **5**. The tip is excited to have a vibration of *A*_t_sin(2*πf*_t_ *t* + *ϕ*_t_) by the mechanical wave *A*_wave_sin(2*πf*_t_ *t* + *ϕ*_wave_) generated from the holder transducer. The sample vibration *A*_s_sin(2*πf*_s_ *t* + *ϕ*_s_) is stimulated by the sample drive via inverse piezoelectric effect. Considering the influence of first‐harmonic electrostatic force *C*′(*V*_dc_ − *V*_cpd_)*V*_s_sin(2*πf*_s_ *t*), which causes a second tip vibration *A*_EF_sin(2*πf*_s_ *t* + *ϕ*_EF_) (including the contributions from both local and distributed electrostatic forces) through the cantilever transfer function *H*_EF_(*ω*). With these three vibration items, the time‐dependent tip‐sample interaction force now becomes

(7) $$\begin{array}{c} F_{\mathrm{ts}}\left(z,t\right)=F_{\mathrm{ts}}\left[z_{0}+A_{t}\sin\left(2\pi f_{t}t+\phi_{t}\right)+A_{\mathrm{EF}}\sin\left(2\pi f_{s}t+\phi_{\mathrm{EF}}\right)\right.\\ \left.-A_{s}\sin\left(2\pi f_{s}t+\phi_{s}\right)\right] \end{array}$$

Applying the same mathematical process of Equation (4) to (6), two difference‐frequency forces controlled by sample vibration [*F*_ts_(*z*)_diff‐s_] and electrostatic force [*F*_ts_(*z*)_diff‐EF_] will be generated, which are respectively given by

(8) $$F_{\mathrm{ts}}{\left(z\right)}_{\mathrm{diff}-s}=-\frac{1}{2}F_{\mathrm{ts}}^{\prime \prime }\left(z_{0}\right)A_{s}A_{t}\cos\left(2\pi f_{\mathrm{diff}}t+\phi_{s}-\phi_{t}\right)$$

(9) $$F_{\mathrm{ts}}{\left(z\right)}_{\mathrm{diff}-\mathrm{EF}}=\frac{1}{2}F_{\mathrm{ts}}^{\prime \prime }\left(z_{0}\right)A_{\mathrm{EF}}A_{t}\cos\left(2\pi f_{\mathrm{diff}}t+\phi_{\mathrm{EF}}-\phi_{t}\right)$$

then the forces *F*_ts_(*z*)_diff‐s_ and *F*_ts_(*z*)_diff‐EF_ drive the cantilever to vibrate at *A*_diff‐s_sin(2*πf*_diff_ *t* + *ϕ*_diff‐s_) and *A*_diff‐EF_sin(2*πf*_diff_ *t* + *ϕ*_diff‐EF_) respectively via cantilever transfer function *H*_ts_(*ω*). Finally, these two difference‐frequency vibrations *A*_diff‐s_sin(2*πf*_diff_ *t* + *ϕ*_diff‐s_) and *A*_diff‐EF_sin(2*πf*_diff_ *t* + *ϕ*_diff‐EF_) vectorially synthesize to the final HM‐PFM DFP signal *A*_diff_sin(2*πf*_diff_ *t* + *ϕ*_diff_). Note that the cantilever transfer functions *H*_wave_(*ω*), *H*_EF_(*ω*), and *H*_ts_(*ω*) are different due to the difference of excitation schemes, which are mechanical wave, electrostatic force (here considering the local and distributed parts by one transfer function for simplification, see Section S3 in the Supporting Information), and local tip‐sample interaction force excitations, respectively. As *A*_diff_sin(2*πf*_diff_ *t* + *ϕ*_diff_) is the vector sum of *A*_diff‐s_sin(2*πf*_diff_ *t* + *ϕ*_diff‐s_) and *A*_diff‐EF_sin(2*πf*_diff_ *t* + *ϕ*_diff‐EF_), *A*_diff_sin(2*πf*_diff_ *t* + *ϕ*_diff_) can be dominated by *A*_diff‐s_sin(2*πf*_diff_ *t* + *ϕ*_diff‐s_) (i.e., the electrostatic force contribution is negligible) if *A*_diff‐s_ is far larger than *A*_diff‐EF_. Since *A*_diff‐s_sin(2*π*f_diff_ *t* + *ϕ*_diff‐s_) and *A*_diff‐EF_sin(2*πf*_diff_ *t* + *ϕ*_diff‐EF_) are stimulated by *F*_ts_(*z*)_diff‐s_ and *F*_ts_(*z*)_diff‐EF_ via an identical cantilever transfer function, *A*_diff‐EF_ and *A*_diff‐s_ are actually governed by the magnitudes of *F*_ts_(*z*)_diff‐s_ and *F*_ts_(*z*)_diff‐EF_. Further comparing the Equations (8) and (9), it is obvious to see that the ratio of these two forces is fundamentally determined by sample voltage‐induced piezoelectric vibration *A*_s_ and the electrostatic force‐induced tip vibration *A*_EF_. As a consequence, by controlling the relative magnitudes of *A*_s_ and *A*_EF_, it is able to make *A*_diff‐s_ far larger than *A*_diff‐EF_ thus to minimize the electrostatic force contribution. Fortunately, for a given sample drive and electrostatic force, *A*_EF_ is governed by the cantilever transfer function *H*_EF_(*ω*) while *A*_s_ is separately determined by the piezoelectric coefficient *d*_33_. Under high‐frequency excitation, due to the significant inertial stiffening and internal damping effect, the transfer function *H*_EF_(*ω*) will dramatically attenuate *A*_EF_ both for on‐ and off‐resonance states,^[^ ^24^, ^26^, ^30^ ^]^ which has been verified by theoretical calculation and experiment (see details in Section S4 in the Supporting Information). On the contrary, the piezoelectric coefficient *d*_33_ is not expected to decrease within MHz frequency band,^[^ ^17^, ^71^ ^]^ indicating that *A*_s_ can almost keep as constant under MHz high‐frequency excitation. This huge difference in the frequency dependences of *H*_EF_(*ω*) and piezoelectric coefficient now allows to significantly reduce *A*_EF_ while keeping *A*_s_ unchanged (i.e., to make *A*_diff‐s_ far larger than *A*_diff‐EF_) by just increasing the excitation frequency (Figure S7, Supporting Information). Typically, in HM‐PFM measurement, the drive frequency of tip *f*_t_ is near the eigenfrequency of a high eigenmode, while the sample drive frequency *f*_s_ is set as *f*_t_ + *f*_diff_, which is usually located within off‐resonance region thus *A*_EF_ will not get the resonance amplification (i.e., *A*_EF_ is attenuated both by high frequency and off‐resonance). Therefore, the HM‐PFM amplitude and phase in Figure 4i,j and Figure S6 in the Supporting Information can keep constant when electrostatic force is largely changed, this is because, under the high‐frequency excitations, *A*_s_ is dominantly larger than *A*_EF_ and the contribution from electrostatic force is negligible.

For comparison, the piezoresponse signal generation mechanism in conventional PFM had also been analyzed in Section S4 in the Supporting Information. Although increasing the excitation frequency to minimize the electrostatic force contribution can also be applied in the conventional PFM,^[^ ^24^, ^30^, ^33^ ^]^ compromise must be made to avoid significant decrease of the sample vibration signal since the transfer functions *H*_EF_(*ω*) and *H*_ts_(*ω*) are correlated (see Figure S5 in the Supporting Information). Meanwhile, the electrostatic force consists of two parts, the distributed part along the whole cantilever and the local part around the tip apex. Increasing drive frequency in the conventional PFM is mainly able to reduce the contribution from the distributed electrostatic force,^[^ ^30^, ^33^ ^]^ thus this method will be effective when the distributed electrostatic force is significant. Whereas if the local part dominates, increasing drive frequency is hard to effectively minimize the electrostatic force contribution due to the correlation of *H*_EF_(*ω*) and *H*_ts_(*ω*) (see details in Section S3 in the Supporting Information). Note that using cantilever with large stiffness has also been extensively proposed to minimize the distributed electrostatic force in conventional PFM,^[^ ^8^, ^25^, ^27^, ^32^ ^]^ but the stiff cantilever associated problems, such as the decrease of sensitivity and large risk of tip‐sample damages, still limit its application. However, the situation is different in HM‐PFM, where both distributed and local electrostatic force contributions can be largely minimized at high frequency due to the heterodyne detection scheme even if the stiffness of the cantilever is small (Figure S7, Supporting Information). Thus, in HM‐PFM, not only effective minimization of electrostatic force can be achieved, but also soft cantilever is recommended for better sensitivity and less tip‐sample damages. In short, introducing heterodyne detection to PFM has changed the generation mechanism of the conventional piezoresponse signal, and breaks the direct coupling between the piezoelectric and electrostatic force signals, which greatly supports to minimize the electrostatic force contribution by using high‐frequency excitation.

### 2.7 Difference‐Frequency Piezoresponse Frequency Spectrum

**Figure 6:**
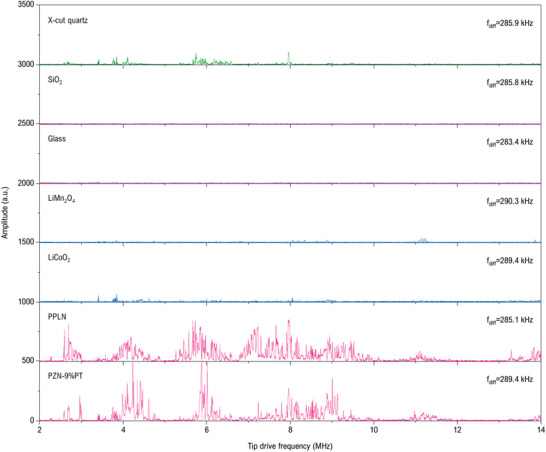
HM‐PFM DFPFS measurements on different materials. Measurement conditions: sample drive, 10 *V*_pp_; holder transducer drive, 12 *V*_pp_; sample drive frequency *f*_s_ = *f*_t_ + *f*_diff_ and *f*_diff_ is set as the first‐order contact resonance frequency of the cantilever (240AC‐PP tip is used) on each sample respectively. Note all the spectrums are offset for clarity.

As the electrostatic force issue has been well addressed in HM‐PFM, then according to the principle of HM‐PFM, if an obvious DFP signal can be observed in HM‐PFM, it will strongly indicate the existence of a true sample vibration induced by electric field. We therefore examine the DFP signal by scanning the drive frequency *f*_t_ and *f*_s_ simultaneously (keep *f*_diff_ constant) to observe the amplitude peaks of the DFP signal. As the tip vibration will get resonance amplification when *f*_t_ is near the eigenfrequency of each eigenmode, then if sample has detectable DFP, multiple amplitude peaks will emerge when *f*_t_ and *f*_s_ are scanned (the peak position is determined by the dynamics of each probe). For instance, if a material has noticeable electromechanical coupling such as piezoelectricity, a series of enhanced peaks should be observed in the DFP spectrum with the frequency scanning, whereas no peaks should be observed if the sample has no electromechanical coupling. Accordingly, we develop this approach as a special HM‐PFM measurement, which is called DFPFS.^[^ ^54^ ^]^ To test the validity of the DFPFS, three types of materials are selected here, including dielectric materials (SiO_2_ and glass), lithium battery materials (LiCoO_2_ and LiMn_2_O_4_) and ferroelectric materials (PPLN and PZN‐9%PT), and the X‐cut quartz single crystal with thickness of 0.5 mm is also tested here as a reference. Before measuring DFPFS, the tip is electrically tuned on each sample to measure the first‐order contact resonance frequency *f*_CR0_ by using conventional PFM. When performing DFPFS measurement, the same tip is used for all samples, the holder drive amplitudes and sample drive amplitudes are set to be uniform for all samples respectively. **Figure** **6** shows the DFPFS measured on the above seven samples with tip frequency from 2 to 14 MHz (*f*_diff_ is set as the *f*_CR0_ on each sample and all the contact resonance curves are shown in Figure S9 in the Supporting Information). Firstly, for the quartz reference sample (with *d*_33_ = 2.3 pm V^−1^),^[^ ^17^ ^]^ its DFPFS shows several observable resonance‐like peaks, indicating that the DFP is detectable on materials with weak piezoelectricity. Conspicuously, the DFPFS of the dielectric and lithium battery samples manifest a dramatic difference with that of the ferroelectric materials. For the ferroelectric samples tested here, many resonance‐like peaks have emerged in the entire of the spectrums which corresponds to a strong DFP. However, there are almost no noticeable (or very weak) peaks shown in the spectrums of dielectric and lithium battery samples, indicating that there is no DFP or the DFP is too weak to be detected. According to the signal generation mechanism of the HM‐PFM, very weak DFP signal implies that the sample vibration is hard to be excited by the applied high‐frequency voltages. For the dielectric glass and SiO_2_, it is easy to understand that there is no apparent first‐order electromechanical coupling in these two materials thus no sample strain and DFP will be excited. Although obvious contact resonance can be observed on SiO_2_ and glass by using the conventional PFM (Figure S9, Supporting Information), this contact resonance actually is induced by electrostatic force instead of electromechanical coupling, thus the DFPFS results obtained here clearly indicate that pure electrostatic force can produce ideal piezoresponse in the conventional PFM but cannot induce noticeable DFP in the HM‐PFM. Note that for the lithium‐ion battery samples, the motion of the Li^+^ in these materials will cause the electrochemical Vegard strain thus it is expected to generate DFP signal if referring to previous studies by electrochemical strain microscopy (ESM).^[^ ^57^, ^60^ ^]^ However, concerns has been raised regarding the veracity of the ESM where the formation of ionic (e.g., Li^+^) concentration gradients is expected to be too slow to contribute to the ESM signal at typical frequencies operated in ESM (≈300 kHz).^[^ ^17^ ^]^ In addition, a newly developed metrological PFM technique with the interferometric displacement sensor PFM (IDS‐PFM),^[^ ^71^ ^]^ which allows to identify and quantify the real sample vibration, has revealed a pretty weak electrochemical strain signal on ceria where the ESM signal is strong.^[^ ^17^ ^]^ Therefore, the true electrochemical strain may be quite small per se under general ≈300 kHz excitation, now this small strain will be further attenuated due to the 10 to 100 times higher frequencies used in the HM‐PFM, hence it is reasonable that the lithium‐ion battery materials tested here show only very weak (or neglectable) DFP signal in the DFPFS measurement. These results indicate that the high‐frequency design in HM‐PFM indeed goes into effect for minimizing both the electrostatic force and electrochemical strain contributions. More importantly, the DFPFS does manifest a distinct characteristic for sample with true electromechanical vibration, thus it can be regarded as powerful evidence when identifying the piezoelectricity of unknown materials.

### 2.8 Difference‐Frequency Piezoresponse of MAPbI_3_ Perovskite

**Figure 7:**
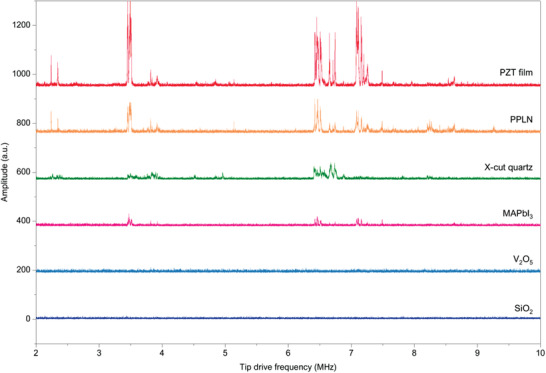
HM‐PFM DFPFS measurements on MAPbI_3_ and reference samples. Measurement conditions: sample drive, 4 *V*_pp_; holder transducer drive, 4 *V*_pp_; sample drive frequency *f*_s_ = *f*_t_ + *f*_diff_ and *f*_diff_ is set as the first‐order contact resonance frequency of the cantilever (PPP‐CONTSCPt tip is used) on each sample respectively. Note all the spectrums are offset for clarity.

With the advanced capabilities of the HM‐PFM, it would be of great interest to apply it to characterize some materials with controversy argument of their piezoelectricity phenomena. One of such a representative is the MAPbI_3_ perovskite. MAPbI_3_ is a hybrid organic–‐inorganic perovskite (HOIP) which have achieved great interest in recent years for high‐efficiency photovoltaic applications.^[^ ^79^ ^]^ However, to date many mysteries of this material still remain to be elucidated, especially the underlying mechanism of the high photovoltaic performance. Driven by the ferroelectric interpretation for high photovoltaic conversion efficiency,^[^ ^80^ ^]^ conventional PFM has recently been extensively utilized to detect the nanoscale ferroelectric signature of MAPbI_3_ as well as other HOIPs, and indeed, the observations of domain‐like piezoresponse distribution and hysteresis loop seems well support the hypothesis of the ferroelectric nature.^[^ ^10^, ^47^, ^51^, ^53^ ^]^ However, due to the increasingly recognized signal source issue of the PFM, some researchers have conducted careful studies on the MAPbI_3_ with PFM and other measurements and a completely contradictory conclusion, i.e., the MAPbI_3_ is non‐ferroelectric, has also been reached.^[^ ^9^, ^11^, ^12^, ^48^, ^52^, ^81^ ^]^ To date, the real ferroic nature of MAPbI_3_ and other HOIPs is still on hot debate. The complexity of this topic mainly stems from two aspects, one is the material itself and another is the PFM technique. For the aspect of materials, the final properties of the MAPbI_3_ samples may vary largely per se due to the differences in substrates, processing conditions and compositions.^[^ ^50^ ^]^ Meanwhile, the ionic migration has been discovered in HOIPs,^[^ ^50^, ^82^ ^]^ implying that the electrochemical Vegard strain may exist in the PFM measurement of this material thus causing significant influence to the piezoresponse signal. Furthermore, the good electronic and ionic conductivity of MAPbI_3_ results in large uncertainties to the electric field poling and hysteresis loop experiments,^[^ ^53^ ^]^ which hampers researchers to acquire further ferroelectric or non‐ferroelectric evidences. Then for the aspect of PFM, the difficulty toward identifying the ferroelectricity of MAPbI_3_ by PFM is undoubtedly caused by the signal source issue, as MAPbI_3_ is exactly such an intractable case that its PFM signal source, probably the piezoelectric strain, electrostatic force‐induced vibration, electrochemical Vegard strain and other potential effects unnoticed by far, cannot be unambiguously determined beforehand. To date, only the results from the IDS‐PFM (almost the most precise PFM technique currently) have shown that the commonly observed piezoresponse of MAPbI_3_ is not dominated by the true electromechanical strain.^[^ ^9^, ^12^, ^17^ ^]^ To further investigate the electromechanical coupling phenomenon of MAPbI_3_, the HM‐PFM DFPFS measurement is implemented. The MAPbI_3_/V_2_O_5_/ITO is used here as the test sample (see sample information in Section S9 of the Supporting Information), and the X‐cut quartz, SiO_2_, PPLN and PZT film are measured concurrently as the reference. In addition, the DFPFS of V_2_O_5_/ITO substrate is also measured here as a control experiment. Using the same DFPFS measurement procedure introduced above, the DFPFS results of MAPbI_3_, V_2_O_5_ and reference samples are obtained and plotted in **Figure** **7**. With using conventional PFM, a remarkable piezoresponse can be observed on MAPbI_3_ sample (Figure S13b, Supporting Information), highly indicating that there may exist an obvious electromechanical coupling like piezoelectricity. In contrast, during the HM‐PFM measurements, as the contributions from electrostatic force and the electrochemical Vegard strain are minimized due to the high‐frequency excitation, the DFPFS of MAPbI_3_ in Figure 7 does show a much weaker DFP than that of the piezoelectric samples including the X‐cut quartz (see more data in Figure S13 of the Supporting Information), and only several very weak peaks can be observed, this can be compared with the DFPFS of the SiO_2_ sample which has no observable DFP peaks. Since the control experiment of the DFPFS on V_2_O_5_ substrate (Figure 7 and Figure S13d in the Supporting Information) shows no noticeable DFP peaks, it can be inferred that there should exist a true but very weak electromechanical coupling in the MAPbI_3_ sample studied here, which is consistent with the speculation of previous IDS‐PFM studies.^[^ ^9^, ^12^, ^17^ ^]^ Note that the MAPbI_3_/V_2_O_5_/ITO sample used here is a thin film sample with MAPbI_3_/V_2_O_5_ thickness of ≈200/10 nm, while the quartz sample has a thickness of 0.5 mm. Therefore, during the DFPFS measurements, the effective electric field in MAPbI_3_ sample is expected to be much higher than that of quartz as the drive amplitudes of the two samples are the same, this implies that this truly existed electromechanical coupling should have a much smaller coupling coefficient than that of the quartz (2.3 pm V^−1^). Although to further identify the origin of this weak electromechanical coupling is out of the scope of this study, the obtained results can already lead to a conclusion that the MAPbI_3_ sample tested here have nearly no noticeable piezoelectricity thereby ferroelectricity. However, due to the possible differences between the prepared MAPbI_3_ and other HOIP samples (in fact, the reported topographies or domain distributions of the MAPbI_3_ samples were varied significantly^[^ ^10^, ^48^, ^49^, ^51^ ^]^), the phenomena observed thereby the conclusion drawn here might not be applied to all cases. Whereas unambiguously, the piezoelectricity or ferroelectricity of a specific HOIP sample can always be examined by performing HM‐PFM DFPFS measurement.

## 3 Conclusion

To summarize, in this study, we have introduced an advanced PFM technique, heterodyne megasonic piezoresponse force microscopy or HM‐PFM, which uses 10^6^ to 10^8^ Hz high‐frequency excitation and heterodyne method to measure the piezoelectric strain at nanoscale. It has been confirmed that the HM‐PFM can provide standard measurements for ferroelectric domain and hysteresis loop, and an effective domain characterization with excitation frequency up to ≈110 MHz has been achieved in our model system. With using high‐frequency excitation and heterodyne scheme, both the contributions from local and distributed electrostatic forces have been significantly minimized in HM‐PFM, thereby offering a stronger support to measure hysteresis loop by using continuous DC method. Meanwhile, the electrochemical Vegard strain is also largely attenuated by using the high frequency excitation, thus the electrochemical artifacts can be effectively reduced when using HM‐PFM to study the piezoelectric or ferroelectric properties, especially for those unknown materials. Hence, the advantages of the HM‐PFM are to obtain the ideal ferroelectric domains, ferroelectric hysteresis loops and operate up to as high as ≈110 MHz, at the same time, the electrostatic force and electrochemical contributions have been significantly minimized. Finally, the special measurement offered by HM‐PFM, i.e., the difference‐frequency piezoresponse frequency spectrum or DFPFS, has been demonstrated and a distinct DFPFS characteristic is observed on materials with piezoelectricity. With DFPFS measurement, a truly existed but very weak electromechanical coupling is observed in MAPbI_3_ test sample. In brief, HM‐PFM has simultaneously minimized the influences from multiple signal sources, thus making the target piezoelectric signal considerably purified. Given the challenges and concerns encountered in the conventional PFM measurements, especially the signal source issue, the advantages of HM‐PFM makes it an excellent candidate for the piezoelectric or ferroelectric studies where conventional PFM measurements are highly controversial. Moreover, HM‐PFM successfully provides an access to high‐frequency piezoelectric vibration, thus it can potentially be used to explore the unknown electromechanical coupling phenomena or ferroelectric switching processes under high‐frequency electric field. As an extension, HM‐PFM can be easily modified to realize the detection of high‐order electromechanical coupling, such as the second‐order coupling of electrostriction (Section S10, Supporting Information).^[^ ^54^ ^]^ Conventional PFM‐based method used to measure the electrostrictive strain may suffer the effects of second‐harmonic electrostatic force and Joule heating,^[^ ^37^, ^40^, ^42^, ^46^, ^65^ ^]^ while both of these two effects can be minimized in the HM‐PFM‐based method thus the measurement is expected to be more unambiguous as well (Section S10, Supporting Information).

## 4 Experimental Section

##### HM‐PFM Setup

The model HM‐PFM system was established on a commercial SPM system (SPA400, Seiko Instruments Inc.). The original probe holder was modified to enable the holder transducer to be excited externally with the RG178 coax cable connected. To effectively drive the cantilever's mechanical vibration at high frequency, the holder transducer should have a high center frequency and the acoustic impedance between the AFM probe base and transducer should be small.^[^ ^83^ ^]^ All the AC excitation signals with frequency less than 30 MHz were generated by the arbitrary waveform generator (Keysight 33522B, Keysight Technologies). For the generation of AC signals with frequency higher than 30 MHz, a programmable direct digital synthesizer (AD9959, Analog Devices Inc.) equipped with a radio‐frequency power amplifier was used. An embedded field programmable gate array (FPGA) controller (NI cRIO‐9064, National Instruments) equipped with one digital acquisition card (NI 9775, National Instruments) was utilized to realize the signal acquisition. DC bias was generated by using a digital source meter (Keithley 2450, Tektronix Inc.), and the superposition of DC bias to AC drive was finished by a bias‐tee. The demodulation of the AC signals was completed by using a lock‐in amplifier (MFLI, Zurich Instruments Ltd.). The home‐made analog multiplier, low pass filter, and instrumentation amplifier were used to generate the reference signal for the DFP signal demodulation and amplification. All the programmable instruments were controlled by the self‐developed HM‐PFM control program based on LabVIEW and LabVIEW FPGA (National Instruments).

##### SPM Characterization

In this study, two types of conductive probes were used: Pt coated probe (240AC‐PP, OPUS) with a force constant of ≈2 N m^−1^ and free resonance frequency of ≈70 kHz; Pt‐Ir coated probe (PPP‐CONTSCPt, Nanosensors) with a force constant of ≈0.2 N m^−1^ and free resonance frequency of ≈25 kHz. All the measurements were conducted by contact AFM mode with tip grounded and in ambient environment with humidity of ≈40%. The drive amplitudes were typically set to 6–20 *V*_pp_ for holder transducer and 2–10 *V*_pp_ for sample. Note that too large sample drive may cause apparent sample damage, especially when sample thickness is small. To obtain a DFP signal with good SNR, the selection of the operation frequency is important. Firstly, the difference frequency (*f*_diff_) was always set as the first or second‐order contact resonance frequency of the cantilever, then the tip drive frequency (*f*_t_) (sample drive frequency *f*_s_ = *f*_t_ + *f*_diff_) was determined by scanning *f*_t_ to a frequency position where DFP amplitude reaches to a peak (i.e., to excite a high eigenmode of the cantilever). Note that although multiple DFP peaks may emerge during the frequency scanning, it was found that not all the peaks can be used to optimize DFP signal as there may exist spurious resonance which can cause the measurement unstable. Therefore, to perform an ideal HM‐PFM measurement, it is recommended to determine the optimized operation frequencies firstly via a standard sample (such as PPLN). When measuring the DFPFS of MAPbI_3_ sample, the light source of the optical microscope was turned off for dark conditions, and the measurements were completed within ≈1 h thus the sample degradation can be negligible.^[^ ^9^ ^]^ For each DFPFS measurement on MAPbI_3_, a topography scanning was performed firstly to determine the proper points for DFPFS measurement and after that, the topography was imaged again to check if any variation in topography had occurred. Note that the topography check could be important, especially for thin film sample, as it is found that abnormal DFPFS results may be observed when topography is changed (usually caused by too large electric field) during the measurement, and the possible reasons are still under investigation.

##### Sample Preparation

Several samples were used for this study for different purposes: A commercially available periodically poled lithium niobate (PPLN) sample was used (AR‐PPLN test sample, Asylum Research, Oxford Instruments, USA), which consists of a 3 mm × 3 mm transparent die with a thickness of 0.5 mm.

The PZN‐9%PT single crystal (supplied by Microfine Materials Technology Pte. Ltd., Singapore) with respective orientations of [100]*^L^*/[010]*^W^*/[001]*^T^* was cut into small pieces and the surface of the samples was polished with SiC papers and alumina powder using water‐cooled polisher. After the polishing processes, the dimension of the samples was ≈4 mm (width) × 4 mm (length) × 0.5 mm (thickness).

SiO_2_ sample was the 300 nm thermal oxide layer on Si^+^ wafer, and the glass sample was the ordinary soda lime glass coverslip with 0.2 mm thickness.

LiCoO_2_ and LiMn_2_O_4_ samples were prepared from the commercially available powders, and the powders were firstly dispersed in ethanol and then coated on SiO_2_/Si^+^ substrate.

The PZT 20/80 film with thickness of 300 nm was grown in SrRuO_3_‐buffered SrTiO_3_ substrate with (001) orientation using pulsed laser deposition (KrF excimer laser, *λ* = 248 nm). The SrRuO_3_ layer (≈50 nm) was firstly deposited on the SrTiO_3_ substrate at a temperature of 680 °C and an oxygen pressure of 15 Pa. Then the PZT layer was grown on top of the SrRuO_3_ layer at a temperature of 600 °C and same 15 Pa oxygen pressure. After growth, the film was cooled to room temperature at 10 °C min^−1^ in an oxygen atmosphere of 1 atm.

For the fabrication of the MAPbI_3_ sample, the indium tin oxide (ITO) glass substrates washed sequentially with detergent, deionized water, acetone, and isopropanol were used. Before spin coating, the ITO glass substrates were treated under UV‐ozone for 30 min. The cleaned ITO substrate was spin coated with a thin layer of V_2_O_5_ film (20 mg mL^−1^ dissolved in H_2_O_2_) at 4000 rpm for 30 s (for control experiment, the V_2_O_5_ film was repeatedly coated 4–5 times to get a thicker layer to avoid damage by external electrical field), and then the samples were annealed in ambient air at 120 °C for 10 min. After the V_2_O_5_ was cooled to room temperature, the precursor solution of perovskite (CH_3_NH_3_I:PbI_2_:PbCl_2_ = 209:581:39 mg, dissolved in 1 mL GBL and DMSO with volume ratio of 7:3) was spin coated onto V_2_O_5_ at 1500 rpm for 15 s and 3500 rpm for 35 s, and then annealed at 105 °C for 15 min. During the spin coating process, 0.8 mL toluene was used as the anti‐solvent for quenching at 10 s before the end of the spin coating. The obtained samples were then characterized by using UV–vis absorption spectroscopy and XRD to confirm the MAPbI_3_, and the characterization results are presented in the Section S9 of the Supporting Information.

##### Statistical Analysis

The experimental results for topography image, HM‐PFM mapping, switching spectroscopies, DC spectroscopies, and DFPFS are shown as raw data and no data pre‐processing and statistical analysis have been used. The film thickness was estimated by fitting the histogram of a 256 × 256 AFM topography image using Gaussian distribution model.

## Conflict of Interest

The authors declare no conflict of interest.

## Supporting information

Supporting Information

## Data Availability

Research data are not shared.
