# Supplementary material for: Nanoscale Ferroelectric Characterization with Heterodyne Megasonic Piezoresponse Force Microscopy
Source: Adv Sci (Weinh). 2021 Feb 15;8(8):2003993. doi: 10.1002/advs.202003993 (PMC8061351; doi:10.1002/advs.202003993)
Supplement: Supplementary file 1 — Supporting Information [file ADVS-8-2003993-s001.pdf]

© 2021 Wiley-VCH GmbH

ADVANCED SCIENCE
Open Access

Supporting Information

for *Adv. Sci.*, DOI: 10.1002/advs.202003993

Nanoscale Ferroelectric Characterization with Heterodyne Megasonic Piezoresponse Force Microscopy

*Qibin Zeng, Hongli Wang, Zhuang Xiong, Qicheng Huang, Wanheng Lu, Kuan Sun, Zhen Fan, Kaiyang Zeng**

WILEY-VCH

© 2021 Wiley-VCH GmbH

Supporting Information

Nanoscale Ferroelectric Characterization with Heterodyne Megasonic Piezoresponse Force Microscopy

*Qibin Zeng, Hongli Wang, Zhuang Xiong, Qicheng Huang, Wanheng Lu, Kuan Sun, Zhen Fan, Kaiyang Zeng**

S1. Frequency Response Analysis for Rectangular Cantilever

Figure S1 shows the normalized force constant k_n and quality factor Q_n as a function of mode number (both are normalized with respect to the first eigenmode). Compared with Q_n, k_n shows a much faster increase with the mode number, indicating that the resonance is more and more difficult to be stimulated with increasing frequency.

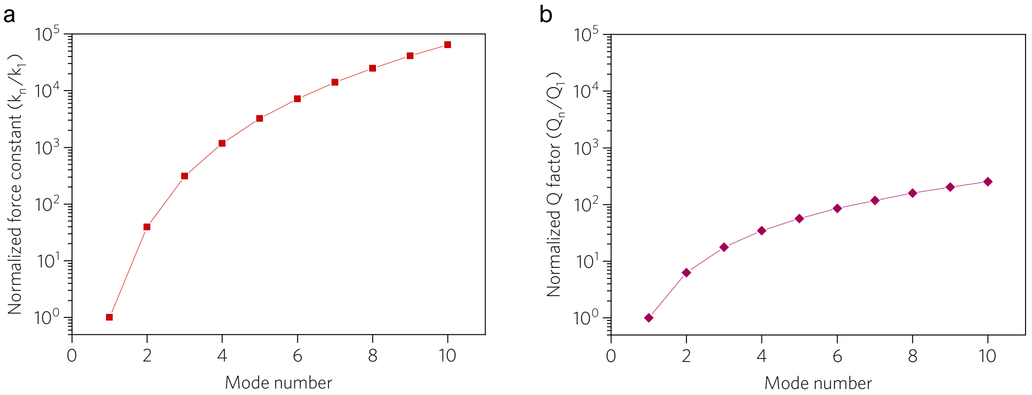

Figure S1. Dependence of force constant and Q factor with mode number. (a) Normalized force constant and (b) normalized Q factor as a function of mode number, both shown logarithmically.

The amplitude response has been calculated here to show the cantilever dynamic property. According to the Euler-Bernoulli beam theory, for a rectangular cantilever, the equation of motion along its longitudinal axis is[1]

$$EI\frac{\partial^4 w(x,t)}{\partial x^4}+c\frac{\partial w(x,t)}{\partial t}+m\frac{\partial^2 w(x,t)}{\partial t^2}=0 \quad \text{(S1)}$$

where E is Young's modulus of the cantilever, I is the moment of inertia, c is the hydrodynamic damping coefficient, m represents the mass per unit length, and $w(x, t)$ is the vertical displacement of the cantilever at the longitudinal position x and time t. Assuming that a point force drive $f(t)$ is applied at the cantilever end ($x = L$), the boundary conditions are given by

$$w(0,t)=0,\ \ w'(0,t)=0,\ \ w''(L,t)=0,\ \ w'''(L,t)=-f(t)/EI \tag{S2}$$

Performing Laplace transform to Equation (S1) and (S2), the motion equation and boundary conditions becomes

$$EI\frac{\partial^4 W(x,s)}{\partial x^4}+\left(cs+ms^2\right)W(x,s)=0 \tag{S3}$$

$$W(0,s)=0,\ \ W'(0,s)=0,\ \ W''(L,s)=0,\ \ W'''(L,s)=-F(s)/EI \tag{S4}$$

The solution of Equation (S3) can be expressed as[2]

$$\begin{gathered} W(x,s)=e^{\lambda x}\left[C_1\sin(\lambda x)+C_2\cos(\lambda x)\right]+e^{-\lambda x}\left[C_3\sin(\lambda x)+C_4\cos(\lambda x)\right] \\ \lambda=\sqrt[4]{\frac{cs+ms^2}{4EI}} \end{gathered} \tag{S5}$$

where C_1, C_2, C_3 and C_4 are undetermined constants. The amplitude response at the cantilever end is defined as

$$A_R(s)=\frac{W(L,s)}{F(s)} \tag{S6}$$

Then using the parameters from AFM tip (240AC-PP, OPUS) and a hydrodynamic damping of 5.06×10^{-4} kg/(m·s)[3] (**Table** S1) to solve the Equation (S3) to (S6) analytically using MATLAB, the amplitude response $A_R(s)$ can be obtained and its magnitude in frequency domain is plotted in **Figure** S2.

Table S1. Parameter values used in the calculation

**Parameter**	*E*	*W*	*h*	*L*	*ρ*	*c*
**Value**	179 GPa	40 μm	2.6 μm	240 μm	2330 kg/m^3	5.6×10^{-4} kg/(m.s)

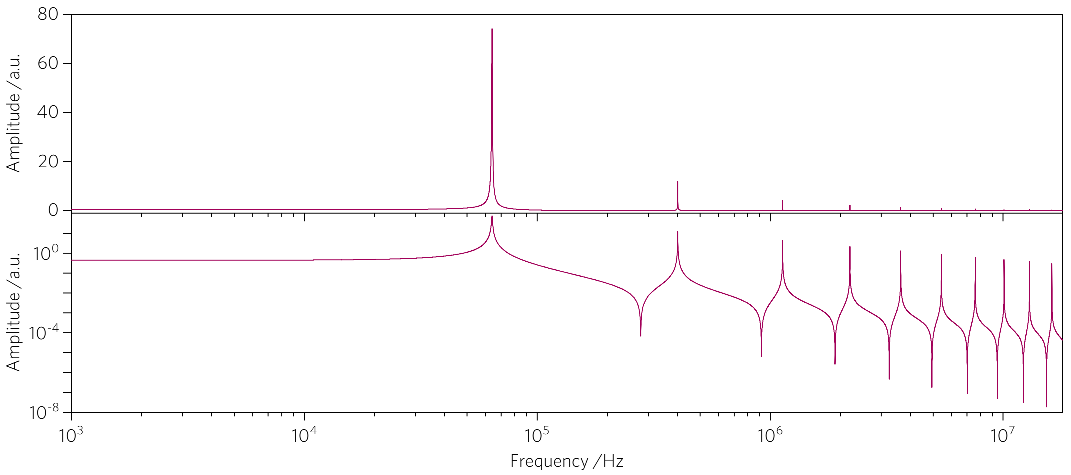

Figure S2. Amplitude response at *x*=*L* as a function of excitation frequency. The amplitude is shown in linear (up) and logarithmic (down) scales respectively.

S2. Heterodyne Detection Principle of HM-PFM

Considering both tip and sample surface have a vibration, $A_t\sin(\omega_t t+\phi_t)$ and $A_s\sin(\omega_s t+\phi_s)$ respectively (here use the angular frequency, $\omega_t = 2\pi f_t$, and $\omega_s = 2\pi f_s$, and similarly hereinafter), the time-dependent tip-sample interaction force is given by

$$F_{ts}(z,t)=F_{ts}\left[z_0 + A_t\sin(\omega_t t+\phi_t) - A_s\sin(\omega_s t+\phi_s)\right] \quad \text{(S7)}$$

Expanding the tip-sample interaction with a Taylor series at $z=z_0$ up to second order gives

$$\begin{aligned} F_{ts}(z,t) &\approx F_{ts}(z_0) + F'_{ts}(z_0)\left(z-z_0\right) + \frac{1}{2}F''_{ts}(z_0)\left(z-z_0\right)^2 \\ &= F_{ts}(z_0) + F'_{ts}(z_0)A_t\sin(\omega_t t+\phi_t) - F'_{ts}(z_0)A_s\sin(\omega_s t+\phi_s) \\ &+\frac{1}{2}F''_{ts}(z_0)A_t^2\sin^2(\omega_t t+\phi_t) + \frac{1}{2}F''_{ts}(z_0)A_s^2\sin^2(\omega_s t+\phi_s) \\ &-F''_{ts}(z_0)A_tA_s\sin(\omega_t t+\phi_t)\sin(\omega_s t+\phi_s) \end{aligned} \quad \text{(S8)}$$

To further process the Equation (S8), the tip-sample interaction force can be expressed by the sum of the components with different frequencies

$$F_{ts}(z,t) \approx F_{ts}(z)_{static} + F_{ts}(z)_{1\omega} + F_{ts}(z)_{2\omega} + F_{ts}(z)_{sum} + F_{ts}(z)_{diff} \quad \text{(S9)}$$

where the $F_{ts}(z)_{static}$, $F_{ts}(z)_{1\omega}$, $F_{ts}(z)_{2\omega}$, $F_{ts}(z)_{sum}$ and $F_{ts}(z)_{diff}$ represent the static, first harmonic, second harmonic, sum-frequency and difference-frequency components of the tip-sample interaction force, respectively, which are given by

$$\begin{cases} F_{ts}(z)_{static} = F_{ts}(z_0) + \frac{1}{4}F''_{ts}(z_0)\left(A_t^2 + A_s^2\right) \\ F_{ts}(z)_{1\omega} = F'_{ts}(z_0)A_t\sin(\omega_t t+\phi_t) - F'_{ts}(z_0)A_s\sin(\omega_s t+\phi_s) \\ F_{ts}(z)_{2\omega} = -\frac{1}{4}F''_{ts}(z_0)A_t^2\cos(2\omega_t t+2\phi_t) - \frac{1}{4}F''_{ts}(z_0)A_s^2\cos(2\omega_s t+2\phi_s) \\ F_{ts}(z)_{sum} = \frac{1}{2}F''_{ts}(z_0)A_tA_s\cos\left[(\omega_t+\omega_s)t+\phi_t+\phi_s\right] \end{cases} \quad \text{(S10)}$$

$$F_{ts}(z)_{diff} = -\frac{1}{2}F''_{ts}(z_0)A_tA_s\cos\left(\omega_{diff}t+\phi_s-\phi_t\right) \quad \text{(S11)}$$

in which $\omega_{diff} = \omega_s - \omega_t$ is the difference frequency. For ferroelectric domains with upward and downward polarization, there is a 180° phase difference between the tip voltage-induced piezoelectric strain, thus the vibration of sample surface has a 180° phase difference between up- and downward domains,[4] which are schematically shown in **Figure** S3. From Equation (S11), when the drive signals applied to the tip and sample remain constant, the difference-frequency tip-sample interaction force on upward and downward ferroelectric domains are calculated by

$$F_{ts}(z)_{diff-down} = -\frac{1}{2}F''_{ts}(z_0)A_tA_s\cos\left(\omega_{diff}t+\phi_s-\phi_t\right) \quad \text{(S12)}$$

$$F_{ts}(z)_{diff-up} = -\frac{1}{2}F''_{ts}(z_0)A_tA_s\cos\left(\omega_{diff}t+\phi_s-\phi_t+180^\circ\right) \quad \text{(S13)}$$

Obviously, there is a theoretical 180° phase difference between $F_{ts}(z)_{diff-up}$ and $F_{ts}(z)_{diff-down}$, implying that

the heterodyne-based PFM has the same capability with conventional vertical PFM with respect to characterizing the polarization of ferroelectric domain.

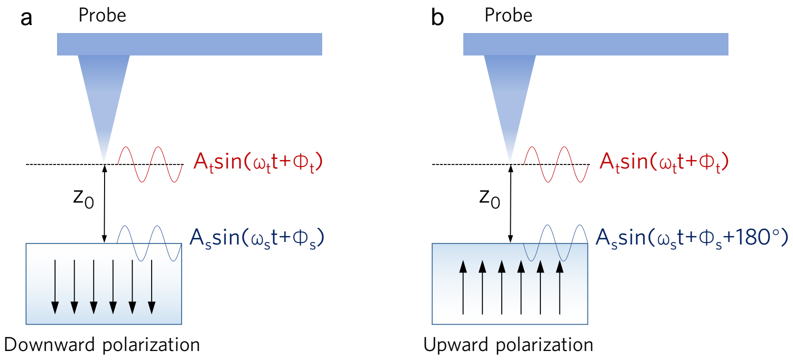

Figure S3. Schematic of the tip-sample interactions on different ferroelectric domains. (a) Dynamic tip-sample interaction on downward and (b) upward polarization domain.

S3. Transfer Functions of Cantilever

To compare the difference of cantilever dynamics under electrostatic force and sample vibration excitations, the cantilever transfer functions have been calculated here. Since the AFM tip is always in contact with sample surface during PFM measurements, the vertical tip-sample coupling is modelled as a spring in parallel with a dashpot (Kelvin-Voigt model) and no lateral contact coupling is considered for simplicity.[5, 6] **Figure** S4 shows the mechanical models used in these calculations. Considering there is only a sample vibration $u(t)$ (**Figure** S4b), the cantilever is driven by the local tip-sample interaction, the equation of motion and the corresponding boundary conditions are:[6]

$$\begin{cases} EI\dfrac{\partial^4 w(x,t)}{\partial x^4}+c\dfrac{\partial w(x,t)}{\partial t}+m\dfrac{\partial^2 w(x,t)}{\partial t^2}=0 \\ w(0,t)=0,\quad w'(0,t)=0,\quad w''(L,t)=0,\quad w'''(L,t)=\dfrac{1}{EI}\left[k_{ts}\left(w(L,t)-u(t)\right)+\gamma\dfrac{\partial\left(w(L,t)-u(t)\right)}{\partial t}\right] \end{cases} \tag{S14}$$

in which k_{ts} and γ are tip-sample contact stiffness and contact damping constant, respectively. Performing Laplace transform to Equation (S14),

$$\begin{cases} EI\dfrac{\partial^4 W(x,s)}{\partial x^4}+\left(cs+ms^2\right)W(x,s)=0 \\ W(0,s)=0,\quad W'(0,s)=0,\quad W''(L,s)=0,\quad W'''(L,s)=\dfrac{(k_{ts}+\gamma s)[W(L,s)-U(s)]}{EI} \end{cases} \tag{S15}$$

and defining the transfer function for sample vibration (the local tip-sample interaction) excitation as

$$H_{ts}(s)=\frac{W(L,s)}{U(s)} \tag{S16}$$

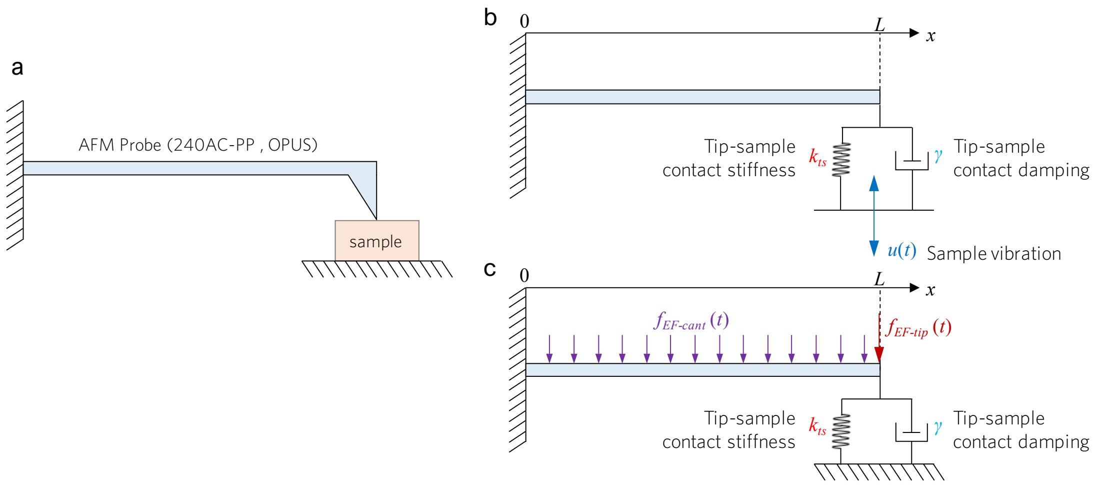

Figure S4. Mechanical models of AFM probe in contact with the sample. (a) Schematic illustration of the AFM probe in contact with the sample. Mechanical models for the calculation of cantilever transfer function with the excitation of (b) sample vibration and (c) electrostatic force.

The solution of Equation (S15) has exactly the same form of Equation (S5), thus by solving Equation (S15), (S5) and (S16) analytically in MATLAB, the transfer function in Laplace domain $H_{ts}(s)$ can be obtained and the frequency domain form $H_{ts}(\omega)$ can be transformed from $H_{ts}(s)$ *via* $s = i\omega$. Using the parameters shown in Table 1 and assuming $k_{ts} = 100k_c = 100\times3EI/L^3$, $\gamma = 0.1\times(EIm)^{0.5}$/L (dimensionless damping constant of 0.1)[7], the calculated transfer function $H_{ts}(\omega)$ is plotted in **Figure** S5.

For the electrostatic force, it is usually considered as the sum of two parts, the distributed part along the whole cantilever $f_{EF\text{-}cant}$ and the local part around the tip apex $f_{EF\text{-}tip}$[4,5,8] (**Figure** S4c), thus under the electrostatic force excitation, the equation of motion and the corresponding boundary conditions now become

$$\begin{cases} EI\dfrac{\partial^4 w(x,t)}{\partial x^4}+c\dfrac{\partial w(x,t)}{\partial t}+m\dfrac{\partial^2 w(x,t)}{\partial t^2}=\dfrac{f_{EF-cant}(t)}{L} \\ w(0,t)=0,\ \ w'(0,t)=0,\ \ w''(L,t)=0,\ \ w'''(L,t)=\dfrac{1}{EI}\left[k_{ts}w(L,t)+\gamma\dfrac{\partial w(L,t)}{\partial t}-f_{EF-tip}(t)\right] \end{cases} \tag{S17}$$

Performing Laplace transform to Equation (S17),

$$\begin{cases} EI\dfrac{\partial^4 W(x,s)}{\partial x^4}+\left(cs+ms^2\right)W(x,s)=\dfrac{F_{EF-cant}(s)}{L} \\ W(0,s)=0,\ \ W'(0,s)=0,\ \ W''(L,s)=0,\ \ W'''(L,s)=\dfrac{(k_{ts}+\gamma s)W(L,s)-F_{EF-tip}(s)}{EI} \end{cases} \tag{S18}$$

and a particular solution of Equation (S18) is $\dfrac{F_{EF-cant}(s)}{L(cs+ms^2)}$,[2] thus the solution of Equation (S18) can be written as

$$W(x,s)=e^{\lambda x}\left[C_1\sin(\lambda x)+C_2\cos(\lambda x)\right]+e^{-\lambda x}\left[C_3\sin(\lambda x)+C_4\cos(\lambda x)\right]+\frac{F_{EF-cant}(s)}{L(cs+ms^2)} \tag{S19}$$

Since the local and distributed electrostatic force are correlated, here for simplicity, a constant ratio α is

introduced to relate $f_{EF\text{-}cant}$ and $f_{EF\text{-}tip}$

$$f_{EF-tip}(s) = \alpha f_{EF-cant}(s), \quad F_{EF-tip}(s) = \alpha F_{EF-cant}(s) \tag{S20}$$

then the transfer function for the electrostatic force excitation can be defined as

$$H_{EF}(s) = \frac{W(L,s)}{F_{EF-cant}(s)} \tag{S21}$$

Using the same parameters and calculation method mentioned above to solve Equation (S18) to (S21), the transfer function in frequency domain $H_{EF}(\omega)$ can be obtained. As the relative magnitude between $f_{EF\text{-}cant}$ and $f_{EF\text{-}tip}$ depends on specific experiment,[4,9] three values of α, 0.001, 0.1 and 10, are used to calculate $H_{EF}(\omega)$ and the results are all plotted in **Figure** S5.

Comparing the transfer function $H_{EF}(\omega)$ (dot line in **Figure** S5) and $H_{ts}(\omega)$ (red solid line in **Figure** S5), it is clear to see that both curves show a decay trend with increasing frequency, while $H_{EF}(\omega)$ obviously decreases faster than $H_{ts}(\omega)$, thus using high frequency in conventional PFM can minimize the contribution of electrostatic force. Furthermore, when α is small, *i.e.,* the electrostatic force is dominated by the distributed part $f_{EF\text{-}cant}$, $H_{EF}(\omega)$ (green dot line) shows a large difference with $H_{ts}(\omega)$ and it decays much faster than $H_{ts}(\omega)$, indicating that high frequency can largely minimize the electrostatic force contribution. However, when α is large, *i.e.,* local part $f_{tip\text{-}cant}$ dominates the electrostatic force, $H_{EF}(\omega)$ (black dot line) is very closed to $H_{ts}(\omega)$, this is because $f_{tip\text{-}cant}$ and sample vibration both belong to local tip-sample interaction excitation. Therefore, if $f_{tip\text{-}cant}$ dominates the electrostatic force, using high frequency in conventional PFM cannot effectively minimize the electrostatic force contribution as the target piezoresponse signal varies synchronously with electrostatic force signal.

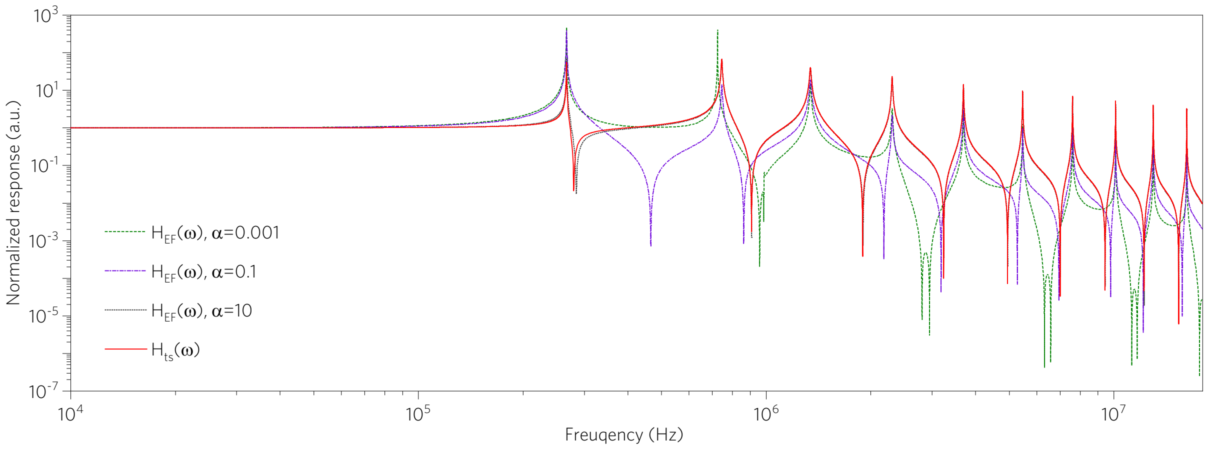

Figure S5. Calculated transfer functions of cantilever. The magnitudes of the transfer functions are all normalized with respect to the low-frequency responses respectively.

S4. Principle of Minimizing the Contribution of Electrostatic Force

X-cut quartz single crystal with thickness of 0.1 mm has been tested here to examine the contribution of electrostatic force in HM-PFM. By scanning DC bias within ±10 V, the amplitude and phase of the difference-frequency piezoresponse (DFP) signal under various excitation frequencies are recorded and the results are all shown in **Figure** S6. Similar with the **Figure 4**i,j in the main text, here all of the amplitude and phase signals also keep constant with changing DC bias, implying that, even on material with weak piezoelectricity, the contribution of electrostatic force can still be neglected in HM-PFM.

The key factor that enables the minimization of electrostatic force contribution in HM-PFM is the difference between frequency dependences of cantilever transfer function $H_{ts}(\omega)$ and piezoelectric strain. Here, still using the same parameters and calculation method described in the S3 of this supporting information, and assuming the total electrostatic force is 10 nN,[4] the amplitude of the electrostatic force excited tip vibration A_{EF} can be calculated. **Figure** S7a shows the calculated A_{EF} as a function of frequency under $\alpha = 0.1$ and 10. For a typical PFM measurement on PPLN sample, the amplitude of the sample vibration A_s is ~10 pm, as the piezoelectric strain does not change apparently within MHz frequency region, the amplitude A_s is schematically plotted by a horizontal line in **Figure** S7a. By comparing A_{EF} and A_s, it is obvious that there exists a huge difference between their frequency dependences, A_{EF} gradually decays with increasing excitation frequency, and in particular, A_{EF} attenuates dramatically at off-resonance or anti-resonance states (such as the shadow area). For low frequency, A_{EF} is larger than A_s, implying that the electrostatic force contribution is significant or even dominant. But at high frequency, by properly choosing the frequency (*e.g.* in the shadow area), A_{EF} (with $\alpha = 0.1$ and 10 both) can be largely attenuated to be much smaller than A_s, thus realizing significant minimization of electrostatic force contribution no matter the electrostatic force is dominated by distributed or local part. Note that the calculation of A_{EF} here is based on multiple assumptions, such as ignoring the internal damping and the frequency dependence of contact damping, the practical attenuation of A_{EF} is much more rapid. **Figure** S7b shows the experimentally measured tip vibration amplitude (the amplitude of cantilever deflection signal) as a function of excitation frequency. This curve is measured on clean SiO_2 surface by conventional PFM set-up with the same AFM tip used for the calculation (*i.e.*, 240AC-PP, OPUS).

As the SiO_2 is the pure dielectric layer of Si wafer, the measured tip vibration is actually stimulated by the electrostatic force. It is obvious that the practical tip vibration excited by electrostatic force decays very fast, just from the 1st to the 3rd eigenmode, the resonant amplitude has already attenuated ~100 times, which highly indicates that at much higher frequency, the practical A_{EF} will further decrease to be far smaller than A_s. Therefore, HM-PFM can achieve almost an ideal minimization of electrostatic force.

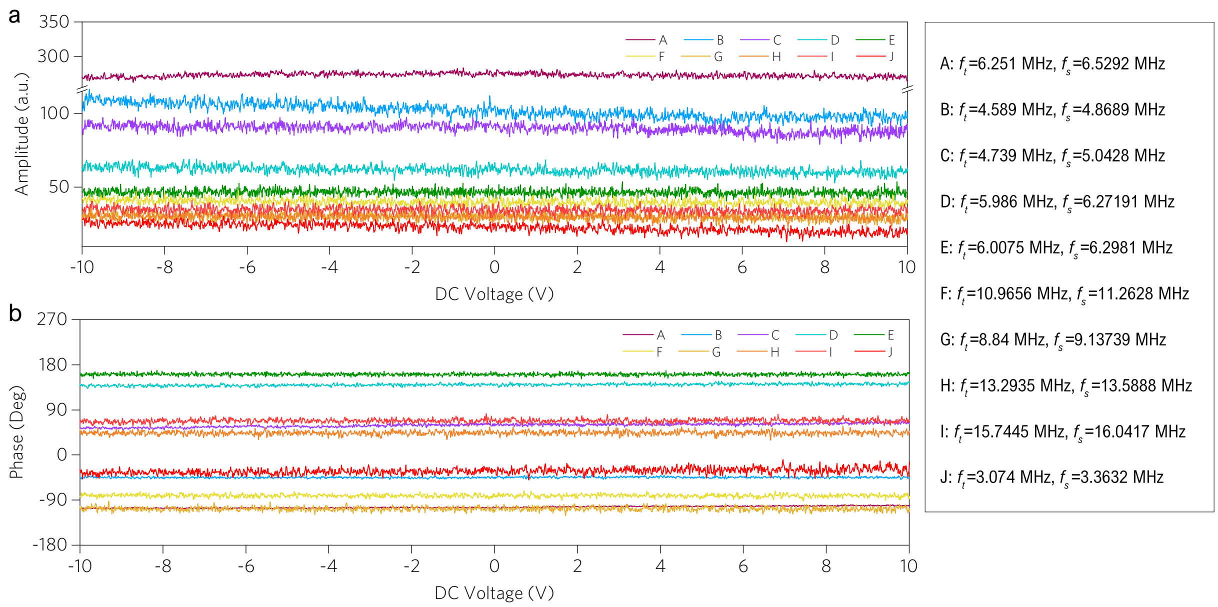

Figure S6. HM-PFM DC spectroscopy of X-cut quartz single crystal. (a) Amplitude and (b) phase of the DFP signal as a function of DC bias under varies drive frequencies.

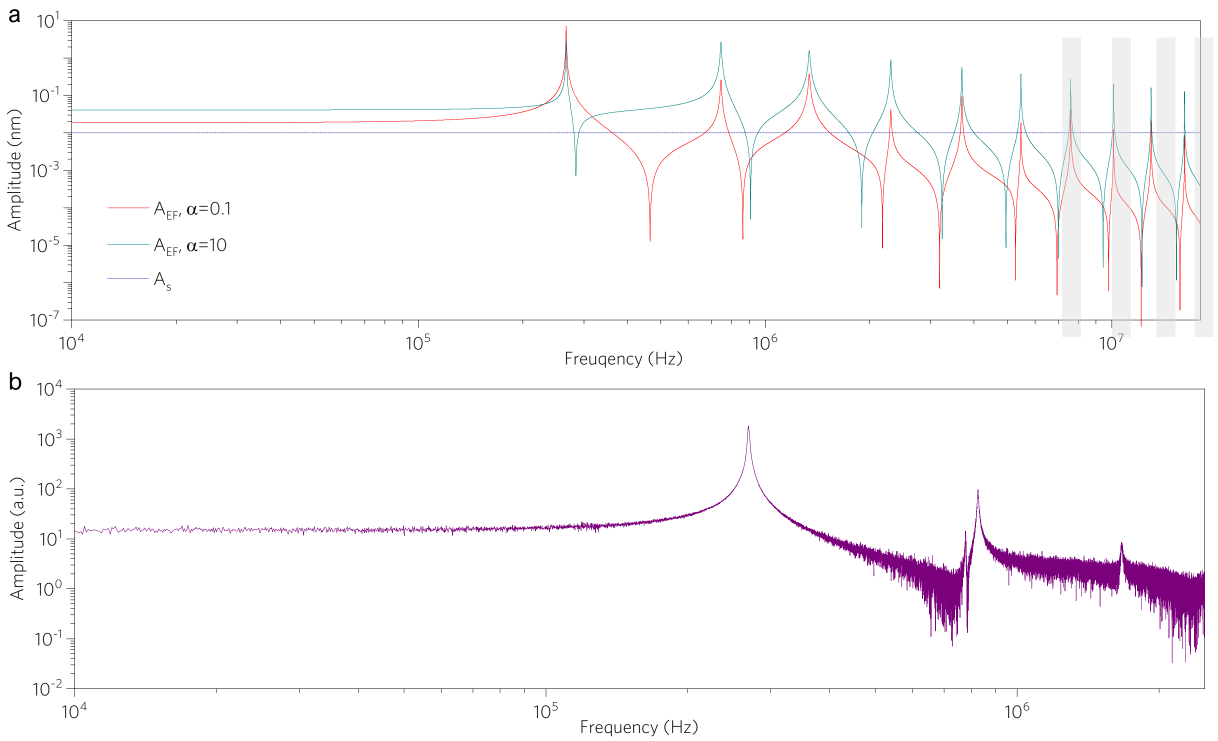

Figure S7. Electrostatic force excited amplitude as a function of excitation frequency. (a) Calculated amplitude A_{EF} (at $x=L$) as a function of excitation frequency, a piezoelectric vibration with amplitude A_s=10 pm is schematically shown by the horizontal dot line. (b) Amplitude of the deflection signal as a function of excitation frequency (measured by single-frequency PFM on SiO_2 sample).

Figure S8 shows that, in the conventional PFM, the tip voltage stimulated piezoelectric vibration will cause a local varying tip-sample interaction force $F_{ts}'(z_0)\sin(\omega_s t+\phi_s)$, this force will drive the cantilever to vibrate and generate the piezoelectric signal $A_{s\text{-}p}\sin(\omega_s t+\phi_{p\text{-}s})$ *via* the cantilever's transfer function $H_{ts}(\omega)$. At the same time, the first harmonic electrostatic force $C'(V_{dc}-V_{cpd})V_{ac}\sin(\omega_s t)$ directly drive the cantilever to vibrate, generating the electrostatic force signal $A_{EF}\sin(\omega_s t+\phi_{p\text{-}EF})$ *via* transfer function $H_{EF}(\omega)$. Finally, the piezoelectric signal $A_{s\text{-}p}\sin(\omega_s t+\phi_{p\text{-}s})$ and electrostatic force signal $A_{EF}\sin(\omega_s t+\phi_{p\text{-}EF})$ will vectorially synthesis to the final piezoresponse signal $A_p\sin(\omega_s t+\phi_p)$. Although multiple methods, such as using probes with large force constant and operating at high frequency or higher eigenmodes,[5, 10] are proposed to minimize the contribution of electrostatic force, these methods in principle are based on the difference between $H_{ts}(\omega)$ and $H_{EF}(\omega)$. However, as shown in **Figure** S5, $H_{ts}(\omega)$ and $H_{EF}(\omega)$ belong to the same cantilever and are correlated internally, it is almost impossible to change $H_{EF}(\omega)$ only while keeping $H_{ts}(\omega)$ unaffected especially when the electrostatic force is dominated by the local part. Therefore, compromise must be made to avoid significant damage to the target piezoelectric signal when addressing the electrostatic force issue in conventional PFM. Similarly, as the Electrochemical Strain Microscopy (ESM) has exactly the same set-up with that of the conventional PFM, the signal generation mechanism discussed above is also applied to ESM where the electrostatic force is also an important issue.[11]

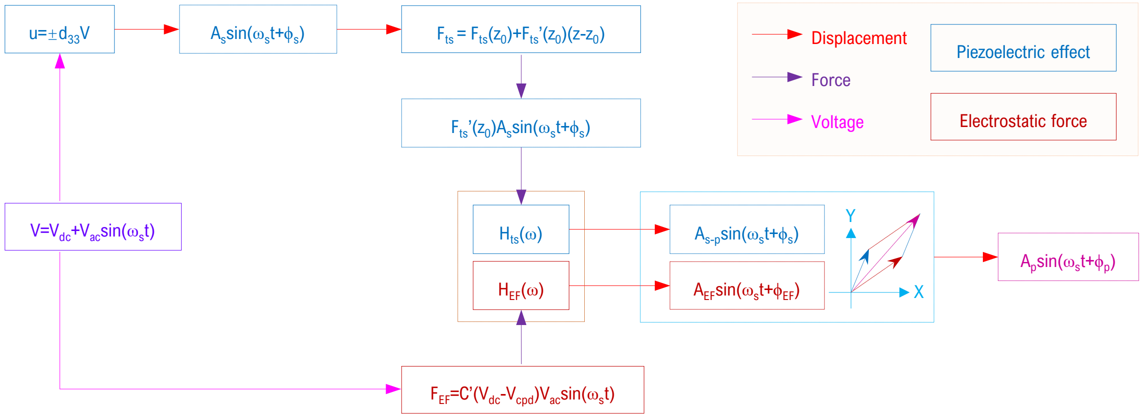

Figure S8. Schematic of piezoresponse signal generation mechanism in conventional PFM.

S5. Contact Resonance Curves Measured by Single-Frequency PFM

Figure S9 shows the contact resonance curves of the 7 samples discussed in the **Figure 6** of the main text, in which are all measured by single-frequency PFM with sample drive amplitude of 2 ~ 10 V_{pp}. The resonance frequencies are all fitted from the resonance curves by using simple harmonic oscillator model.[12]

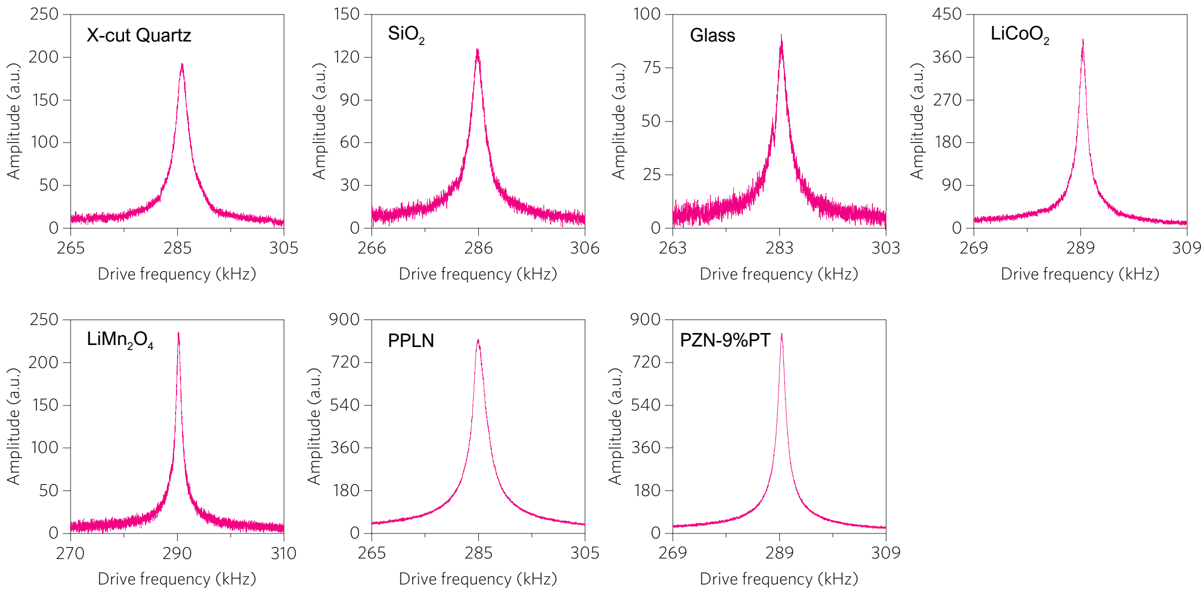

Figure S9. Contact resonance curves measured by single-frequency PFM.

S6. Influence of the Radio-Frequency Radiation

With increasing the excitation frequency of the holder transducer, the radio-frequency radiation around the transducer increases, causing an additional electric field E_{RF} between the tip and substrate. This radiated electric field E_{RF} has the same frequency with the transducer drive. Assuming that E_{RF} is caused by an equivalent AC voltage $V_{RF}\sin(\omega_t t)$ applied between tip and substrate, the total voltage between tip and substate is (ignoring the DC bias and phase here):

$$V_{total} = V_s \sin(\omega_s t) + V_{RF} \sin(\omega_t t) \tag{S22}$$

Then the total electrostatic force under the radiation is given by:

$$F_{EF}^* = \frac{1}{2} C' \left(V_{total} - V_{cpd} \right)^2 \tag{S23}$$

Substituting Equation (S22) into (S23) and rearranging:

$$\begin{aligned} F_{EF}^* = & \frac{1}{2} C' \left(V_{cpd}^2 + \frac{1}{2} V_s^2 + \frac{1}{2} V_{RF}^2 \right) \\ & - \frac{1}{2} C' V_{cpd} \left[V_s \sin(\omega_s t) + V_{RF} \sin(\omega_t t) \right] \\ & - \frac{1}{4} C' \left[V_s^2 \cos(2\omega_s t) + V_{RF}^2 \cos(2\omega_s t) \right] \\ & - \frac{1}{4} C' V_s V_{RF} \cos\left[(\omega_s + \omega_t) t \right] \\ & + \frac{1}{4} C' V_s V_{RF} \cos(\omega_{diff} t) \end{aligned} \tag{S24}$$

From Equation (S24), it is evident that there is a difference-frequency component in F_{EF}^*, $C'V_sV_{RF}\cos(\omega_{diff}t)/4$, which has the same frequency with DFP signal thus it can drive the cantilever and affect the HM-PFM results. However, if the radiation is effectively shielded, V_{RF} will be closed to zero

thus the contribution from the difference-frequency electrostatic force can be neglected.

S7. HM-PFM Measurement on Rough Surface

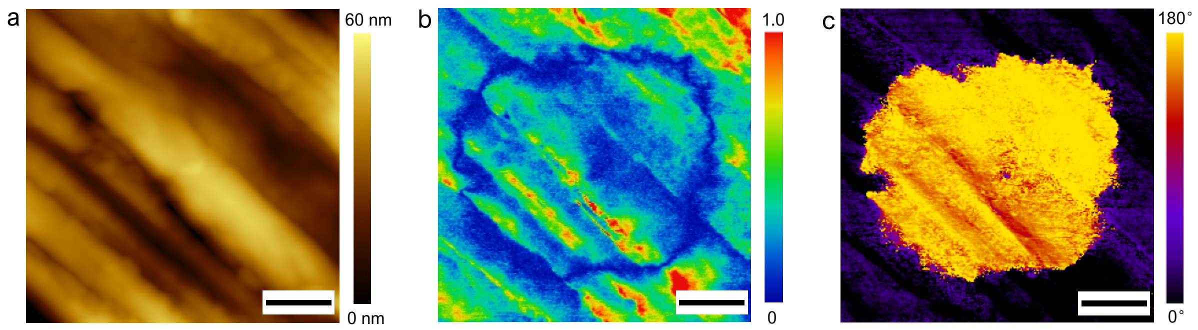

Figure S10. HM-PFM characterization of rough PZN-9%PT sample. (a) Topography, (b) amplitude and (c) phase images of PZN-9%PT with an artificially written domain measured by HM-PFM. Measurement conditions: f_t = 6.128 MHz and f_s = 6.3262 MHz. Scale bar in (a-c), 400 nm and image size = 2 × 2 μm^2.

S8. Influence of Tip-Sample Contact Radius

To check if the HM-PFM result is apparently affected by the tip-sample contact radius, a continuously repeated scanning experiment has been performed. As during continuous tip-sample contact scanning, the tip-sample contact area is expected to be increased due to the tip wearing. **Figure** S11 shows the HM-PFM amplitude and phase images recorded in the repeated scanning on the same area with fixed measurement conditions. It can be seen that from the 1st scanning to the 12th scanning, the spatial resolution is decreased due to the increasing tip-sample contact radius, but the HM-PFM amplitude and phase signals almost keep constant, indicating that the tip-sample contact radius has negligible influence to the HM-PFM signal.

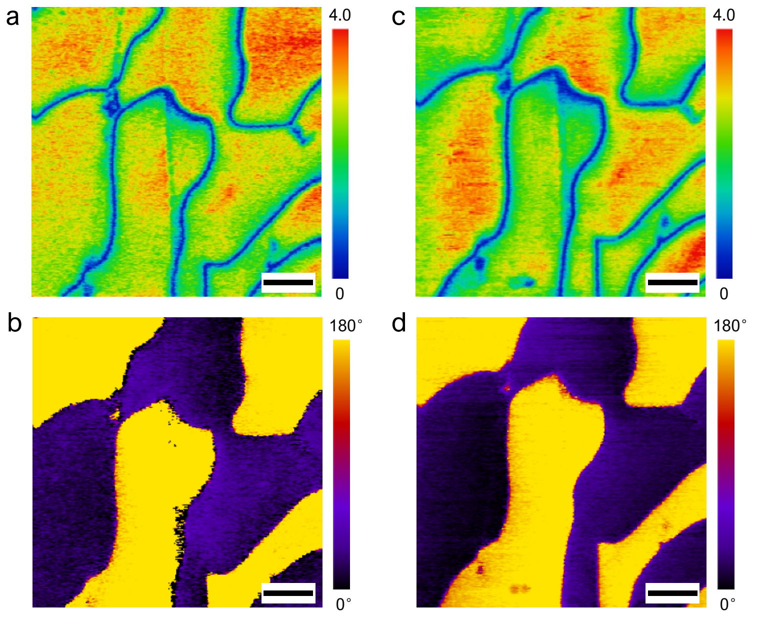

Figure S11. HM-PFM images obtained by continuously repeated scanning. (a, c, e) HM-PFM amplitude images acquired in the 1st, the 6th and the 12th scanning, respectively. (b, d, f) The HM-PFM phase images corresponding to (a), (c) and (e), respectively. Measurement conditions: f_t = 7.09 MHz and f_s = 7.3436 MHz. Scale bar in (a-f), 1 μm and image size = 5 × 5 μm^2.

S9. Supplementary Results of $MAPbI_3$ Perovskite

The fabricated $MAPbI_3$ film has been verified by performing optical absorption and X-Ray Diffraction (XRD) experiments. **Figure** S12a and S12b show the obtained UV-vis absorption spectra and XRD pattern, respectively, which confirms that the sample is $MAPbI_3$ perovskite.[13]

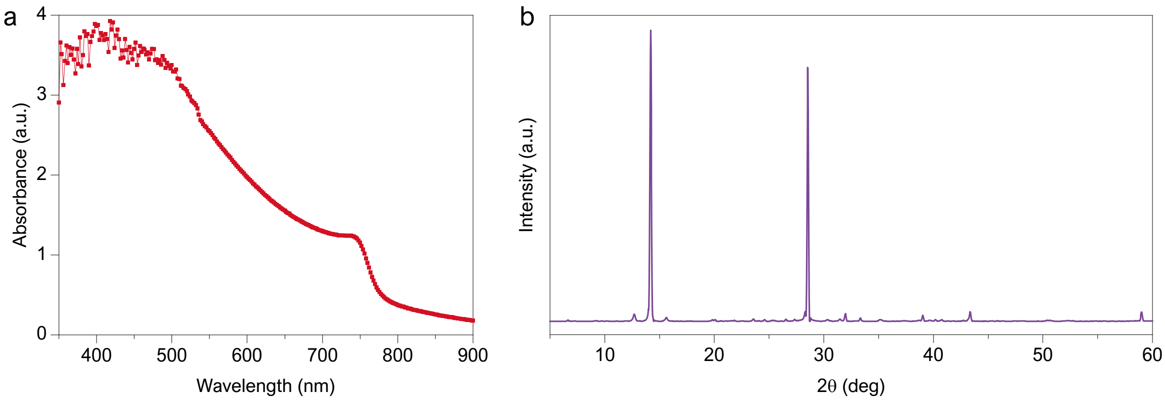

Figure S12. Optical and XRD characterization of $MAPbI_3$ sample. (a) The UV-vis absorption spectra and (b) XRD pattern of the fabricated $MAPbI_3$ film.

Figure S13a shows a typical topography image of the $MAPbI_3$ sample, and the red point indicates a representative position selected for performing the spectrum measurements by conventional PFM and

HM-PFM. Since the $MAPbI_3$ sample is prepared on V_2O_5/ITO substrate, the control experiment has also been performed on blank V_2O_5/ITO sample (to avoid strong electric field-induced sample damage, the V_2O_5 film coated here is ~40 nm thick) to check any potential influences from the V_2O_5 film. The DFPFS of V_2O_5 film measured from the control experiment is shown in **Figure** S13d, and the result indicates that V_2O_5 almost has no electromechanical response thus its influence on the DFPFS measurement of the $MAPbI_3$ can be neglected. Note that the supplementary DFPFS data in **Figure** S13c and S13d uses the same amplitude scale with the **Figure** 7 of the main text.

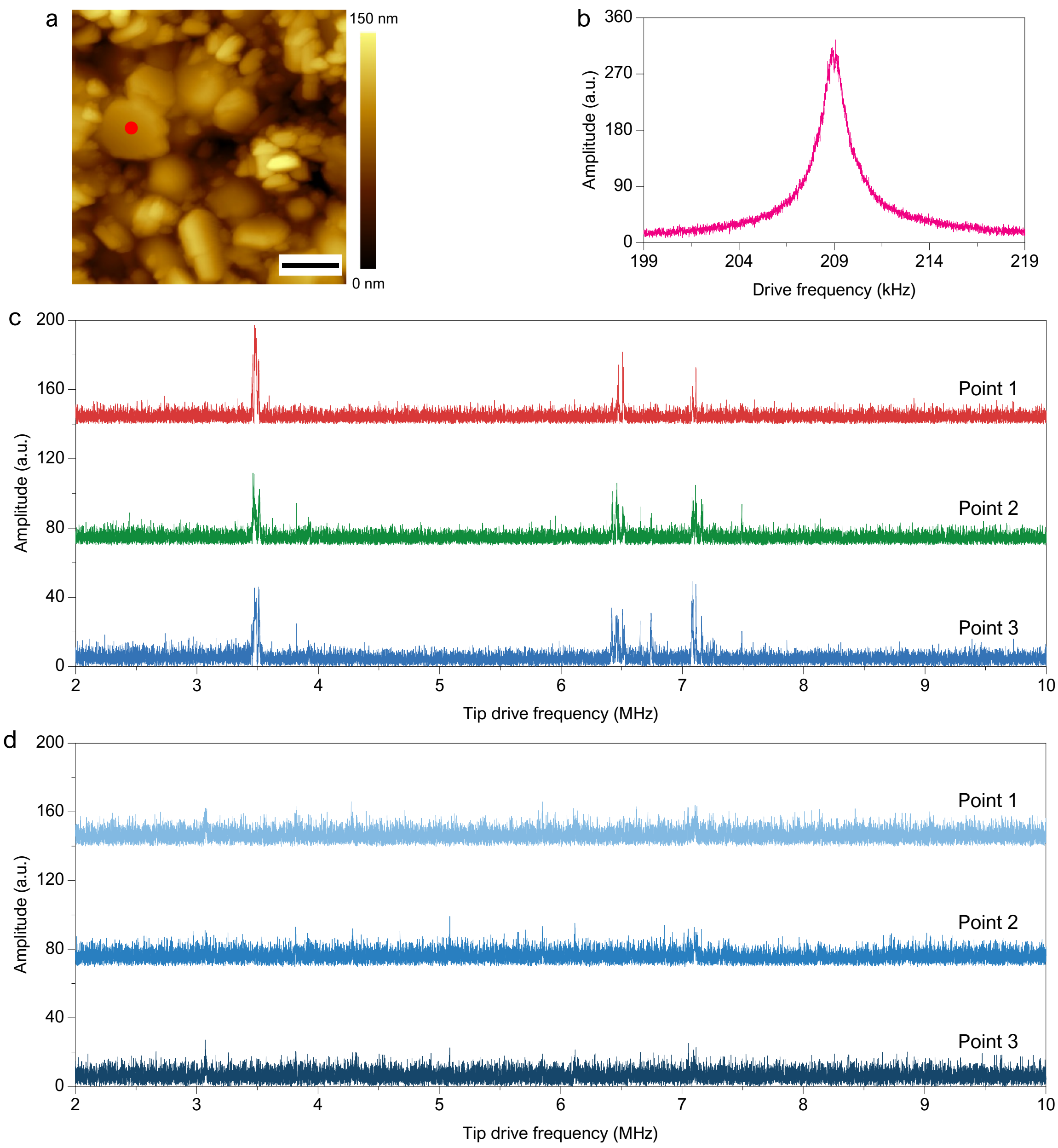

Figure S13. Supplementary data of $MAPbI_3$ sample. (a) A typical topography image of the $MAPbI_3$ sample. (b) The frequency response curve of $MAPbI_3$ measured by conventional PFM (sample drive: 2 V_{pp}). (c) The DFPFS of $MAPbI_3$ measured from different positions. (d) The DFPFS of V_2O_5 film measured from control experiment. Note all the spectrums in (c) and (d) are offset for clarity. Scale bar in (a), 400 nm and image size = 2 × 2 μm^2.

S10. Measurement of Electrostriction

The HM-PFM developed here can be easily modified to measure the high-order electromechanical coupling, such as the important 2nd-order coupling of electrostriction. Conventional PFM-based method has been extensively used to study the electrostriction,[14] while this method is typically influenced by the 2nd-harmonic electrostatic force and Joule heating. Obviously, the 2nd-harmonic electrostatic force contribution can be largely minimized in HM-PFM-based method by similar means of the 1st-harmonic electrostatic force. Then due to the periodical temperature variation decays with increasing frequency,[15] the Joule heating-induced thermal strain can also be diminished in HM-PFM-based measurement. **Figure** S14 shows the modified HM-PFM set-up for the measurement of electrostriction,[16] in which the main modification is the reference signal generation circuit. However, if the reference signal is provided internally by synchronizing the clocks of signal source and lock-in amplifier, no hardware change is needed. As the electrostriction is a quadratic effect, the target electrostrictive vibration locates at the 2nd-harmonic of the sample drive. Therefore, after heterodyne process, the electrostrictive vibration information i s included in the cantilever deflection signal with frequency of $2f_s - f_t$. In a similar fashion, the 3rd-order electromechanical coupling information should reside in the signal with frequency of $3f_s - f_t$ and the nth-order is in $nf_s - f_t$. If the clocks of signal source and lock-in amplifier can be synchronized, then detecting these high-order electromechanical couplings by HM-PFM-based method will be quite straightforward.

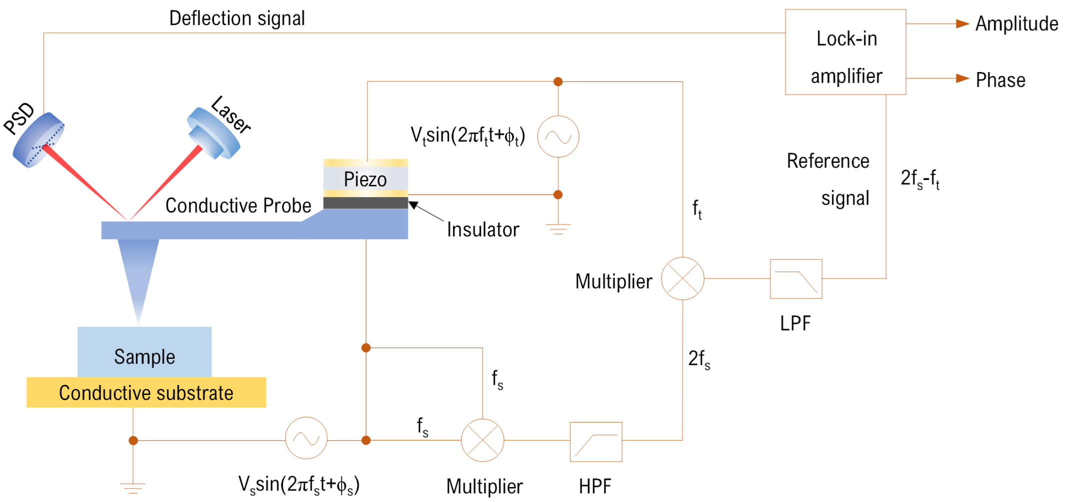

Figure S14. Schematic of the modified HM-PFM set-up for the measurement of electrostriction.

References

[1] G. J. Verbiest, T. H. Oosterkamp, M. J. Rost, *Ultramicroscopy* **2013**, *135*, 113.

[2] F. J. Rubio-Sierra, R. Vazquez, R. W. Stark, *IEEE Trans. Nanotechnol.* **2006**, *5*, 692.

[3] G. J. Verbiest, M. J. Rost, *Ultramicroscopy* **2016**, *171*, 70.

[4] S. V. Kalinin, D. A. Bonnell, *Phys. Rev. B* **2002**, *65*, 125408.

[5] G. A. MacDonald, F. W. DelRio, J. P. Killgore, *Nano Futures* **2018**, *2*, 015005.

[6] U. Rabe, K. Janser, W. Arnold, *Rev. Sci. Instrum.* **1996**, *67*, 3281.

[7] P. A. Yuya, D. C. Hurley, J. A. Turner, *J. Appl. Phys.* **2011**, *109*, 113528.

[8] S. Jesse, A. P. Baddorf, S. V. Kalinin, *Nanotechnology* **2006**, *17*, 1615.

[9] Y. Sugawara, L. Kou, Z. Ma, T. Kamijo, Y. Naitoh, Y. Jun Li, *Appl. Phys. Lett.* **2012**, *100*, 223104.

[10] a) A. Gomez, T. Puig, X. Obradors, *Appl. Surf. Sci.* **2018**, *439*, 577; b) S. Kim, D. Seol, X. Lu, M. Alexe, Y. Kim, *Sci. Rep.* **2017**, *7*, 41657; c) K. Seal, S. Jesse, B. J. Rodriguez, A. P. Baddorf, S. V. Kalinin, *Appl. Phys. Lett.* **2007**, *91*, 232904.

[11] a) V. Lushta, S. Bradler, B. Roling, A. Schirmeisen, *J. Appl. Phys.* **2017**, *121*, 224302; b) N. Balke, S. Jesse, B. Carmichael, M. B. Okatan, I. I. Kravchenko, S. V. Kalinin, A. Tselev, *Nanotechnology* **2017**, *28*, 065704.

[12] S. Jesse, P. Maksymovych, S. V. Kalinin, *Appl. Phys. Lett.* **2008**, *93*, 112903.

[13] a) X. Hou, Y. Hu, H. Liu, A. Mei, X. Li, M. Duan, G. Zhang, Y. Rong, H. Han, *J. Mater. Chem. A* **2017**, *5*, 73; b) X. Guo, C. McCleese, C. Kolodziej, A. C. S. Samia, Y. Zhao, C. Burda, *Dalton Trans.* **2016**, *45*, 3806.

[14] a) Q. N. Chen, Y. Ou, F. Ma, J. Li, *Appl. Phys. Lett.* **2014**, *104*, 242907; b) J. Yu, E. N. Esfahani, Q. Zhu, D. Shan, T. Jia, S. Xie, J. Li, *J. Appl. Phys.* **2018**, *123*, 155104; c) B. Chen, T. Li, Q. Dong, E. Mosconi, J. Song, Z. Chen, Y. Deng, Y. Liu, S. Ducharme, A. Gruverman, F. D. Angelis, J. Huang, *Nat. Mater.* **2018**, *17*, 1020; d) Y. Kim, A. Kumar, A. Tselev, I. I. Kravchenko, H. Han, I. Vrejoiu, W. Lee, D. Hesse, M. Alexe, S. V. Kalinin, S. Jesse, *ACS Nano* **2011**, *5*, 9104.

[15] a) K. Kim, J. Chung, G. Hwang, O. Kwon, J. S. Lee, *ACS Nano* **2011**, *5*, 8700; b) X. Xie, K. L. Grosse, J. Song, C. Lu, S. Dunham, F. Du, A. E. Islam, Y. Li, Y. Zhang, E. Pop, Y. Huang, W. P. King, J. A. Rogers, *ACS Nano* **2012**, *6*, 10267.

[16] K. Zeng, Q. Zeng, *SG. Appl. No. 10202003740V*, **2020**.
